# The health and economic impact and cost effectiveness of interventions for the prevention and control of overweight and obesity in Kenya: a stakeholder engaged modelling study

**DOI:** 10.1186/s12962-023-00467-3

**Published:** 2023-09-21

**Authors:** Mary Njeri Wanjau, Lucy W. Kivuti-Bitok, Leopold N. Aminde, J. Lennert Veerman

**Affiliations:** 1 School of Medicine & Dentistry, Griffith University, Gold Coast campus, Parklands Drive, Southport, Queensland, QLD 4222 Australia; 2 School of Nursing Sciences, University of Nairobi, P.O. Box 19676-00200, Nairobi, Kenya; 3 Non-communicable Disease Unit, Clinical Research Education Networking & Consultancy, Douala, Cameroon

**Keywords:** High body mass, Overweight, Obesity, Non-communicable disease, Costs, Health impact, Economic impact, Cost effectiveness, Kenya, Low- and middle-income

## Abstract

**Background:**

The global increase in mean body mass index has resulted in a substantial increase of non-communicable diseases (NCDs), including in many low- and middle-income countries such as Kenya. This paper assesses four interventions for the prevention and control of overweight and obesity in Kenya to determine their potential health and economic impact and cost effectiveness.

**Methods:**

We reviewed the literature to identify evidence of effect, determine the intervention costs, disease costs and total healthcare costs. We used a proportional multistate life table model to quantify the potential impacts on health conditions and healthcare costs, modelling the 2019 Kenya population over their remaining lifetime. Considering a health system perspective, two interventions were assessed for cost-effectiveness. In addition, we used the Human Capital Approach to estimate productivity gains.

**Results:**

Over the lifetime of the 2019 population, impacts were estimated at 203,266 health-adjusted life years (HALYs) (95% uncertainty interval [UI] 163,752 − 249,621) for a 20% tax on sugar-sweetened beverages, 151,718 HALYs (95% UI 55,257 − 250,412) for mandatory kilojoule menu labelling, 3.7 million HALYs (95% UI 2,661,365–4,789,915) for a change in consumption levels related to supermarket food purchase patterns and 13.1 million HALYs (95% UI 11,404,317 − 15,152,341) for a change in national consumption back to the 1975 average levels of energy intake. This translates to 4, 3, 73 and 261 HALYs per 1,000 persons. Lifetime healthcare cost savings were approximately United States Dollar (USD) 0.14 billion (USD 3 per capita), USD 0.08 billion (USD 2 per capita), USD 1.9 billion (USD 38 per capita) and USD 6.2 billion (USD 124 per capita), respectively. Lifetime productivity gains were approximately USD 1.8 billion, USD 1.2 billion, USD 28 billion and USD 92 billion. Both the 20% tax on sugar sweetened beverages and the mandatory kilojoule menu labelling were assessed for cost effectiveness and found dominant (health promoting and cost-saving).

**Conclusion:**

All interventions evaluated yielded substantive health gains and economic benefits and should be considered for implementation in Kenya.

**Supplementary Information:**

The online version contains supplementary material available at 10.1186/s12962-023-00467-3.

## Background

Globally, non-communicable diseases (NCDs) are the leading cause of deaths (74% of global deaths) and morbidity (64% of disability adjusted life years [DALYs]) [1, 2]. Overweight (Body mass index [BMI] of 25.0-29.9 kg/m^2^) and obesity (BMI ≥ 30.0 kg/m^2^) have been identified as leading risk factors for NCDs [3–5]. The global increase in mean BMI [6, 7] has contributed to a substantial increase of this NCD burden, including in many low- and middle-income countries (LMICs) countries such as Kenya [2, 8, 9]. An estimated 27% of the adult population in Kenya has overweight or obesity (38.5% women [~ 5 million] and 17.5% men [~ 2.2 million]) [10]. The increased BMI related NCD burden greatly impacts individuals’ economic livelihoods and strains the country’s health care system that is still battling communicable, maternal, neonatal and other nutritional diseases, which although in decline, still dominate Kenya’s disease burden [1]. If the current trends continue unabated, it will lead to increased rise in the NCD burden putting further strain on the health system. In our recent study, we found that over the lifetime of the 2019 Kenyan population, high BMI could cause losses of approximately 83.5 million health-adjusted life years (HALYs) (~ 1.7 HALYs per person) and decrease health-adjusted life expectancy by 2.3 years for females and 1.0 years for males [11]. The magnitude of the avoidable high BMI-related disease burden underscores the need to prioritise the control and prevention of overweight and obesity.

In Kenya and other Sub-Saharan Africa countries, high BMI has been linked to changes in dietary patterns and nutrient intakes [12, 13]. These changes are fuelled by factors such as urbanisation, increased income, and changes in the food systems that have seen the expansion of transnational food and drink corporations into ‘emerging markets’ [12–15]. The changes are characterised by a departure from indigenous foods that are often high in carbohydrate, fibre and low in fat and sugar. The indigenous foods are replaced with ultra-processed products, foods high in saturated fat, sugar and salt, low in fibre and other key nutrients [12, 13, 17]. In order to meet global and national obesity reduction targets [17–19], creating healthy environments that stimulate physical activity and encourage a healthy diet may be more impactful than targeting individual behaviour [20, 21]. Policy options directed at the ‘obesogenic’ environment complement education campaigns and social marketing [22]. Evaluation of such policies or preventive interventions enables the identification of those that are impactful and cost effective [23, 24]. This informs judgments on the allocation of resources and priority setting for health. In public health, measurement of outcomes is challenging since most health effects of behaviour change occur only after many years. As a practical alternative to direct measurement, estimates of health outcomes can be obtained through epidemiological modelling [23, 25].

In this study, we assessed interventions for the prevention and control of overweight and obesity in Kenya to determine their potential health and economic impact and cost effectiveness. Intervention selection was informed by a stakeholder engagement process with policy makers in Kenya [26]. To our knowledge, no studies have assessed the potential economic and health impact and cost effectiveness of interventions that aim to prevent and control overweight and obesity in adults in Kenya.

## Methods

### Overview

**Fig. 1:**
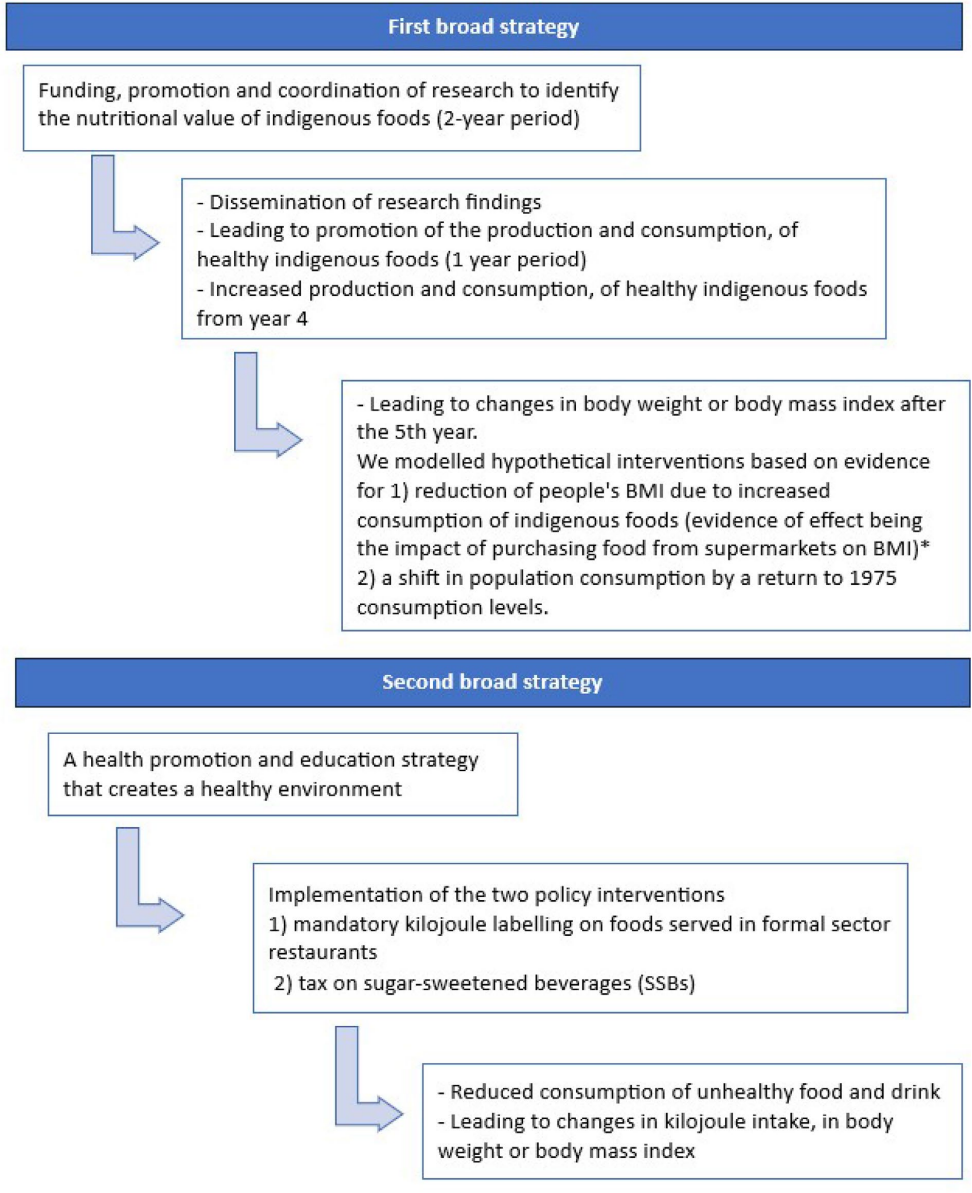
Mapping out implementation scenarios for the selected interventions *Supermarket purchase is seen to contribute to a shift from indigenous foods to processed foods and drinks [40, 41]

**Fig. 2:**
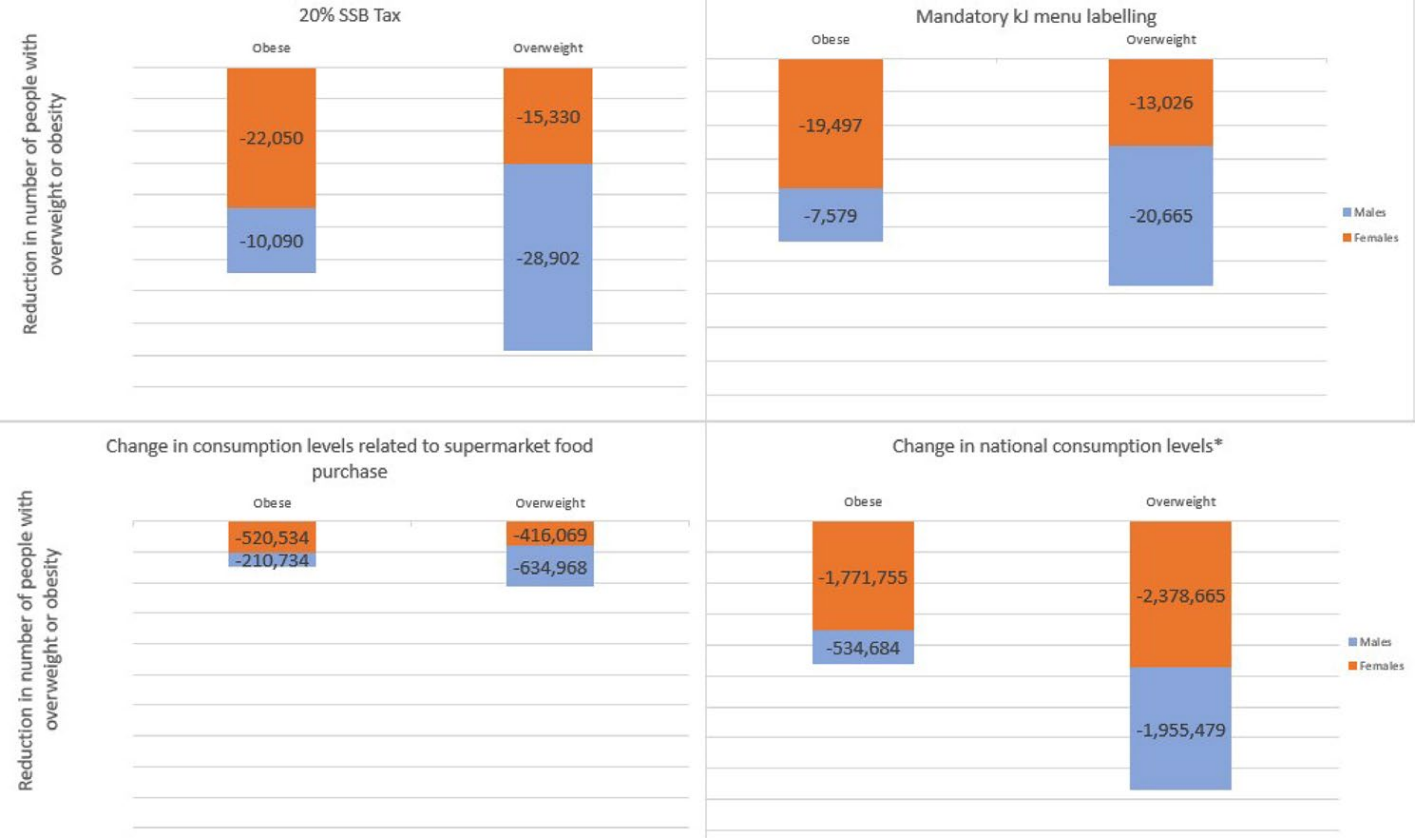
Reduction in number of people with overweight or obesity kJ: kilojoule, BMI: body mass index, SSB: sugar sweetened beverage. *Change in national consumption levels: Change in national consumption levels back to the 1975 average levels of energy intake

In this study, we applied the assessing cost-effectiveness (ACE) approach which has been developed as a priority setting tool that enables evidence-based decision making [27, 28]. Stakeholder engagement was part of the due process and this has been reported elsewhere [26]. We used a lifetime horizon in our assessment and targeted the 2019 Kenya population modelled over their remaining lifetime. We explicitly modelled 37 high BMI related NCDs. We used the WHO recommended generalised cost-effectiveness analysis approach where we compared interventions against a ‘do nothing’ scenario [29]. With respect to body mass, in Kenya, the ‘do nothing scenario’ may also closely reflect the ‘current practice’ in the model 2019 base year. This is because most of the government policy actions on prevention of overweight and obesity are still at the development stage [30]. This comparator scenario is modelled as 2019 BMI levels in Kenya with a linear upward trend continued unabated for 25 years. Baseline BMI levels are taken from published national survey results [10, 31]. We derived the BMI trend from age- and sex-specific mean BMI data for Kenya from 1975 to 2016 as provided in the NCD Risk Factor Collaboration (NCD-RisC) study whose primary data sources are Kenya national surveys done over the years [7]. We checked the BMI levels from the NCD- RisC study (mean and lower, upper uncertainty intervals) against the measured mean (and standard deviation) BMI levels in Kenya from 1993 to 2015 national survey data [31–36] and found the data comparable. In Supplementary File (SF) Fig. 1 we illustrate this using the 2015 BMI data from the two sources [7, 31]. We fitted both the linear and second order polynomial equations trends to the NCD-RisC data and found the results comparable with the projected rise in BMI being slightly lower when the linear trend was applied. The linear trend equations were subsequently used to derive the specific mean BMI for our base year 2019 from 2015 Kenya ‘STEPwise approach to Surveillance of NCD risk factors’ (STEPS) survey data and to predict future mean BMI. Consistent with shifts observed in the work done by Fogel [37], the standard deviation was considered to shift in equal proportion to the mean (i.e., a ratio of 1:1 is assumed). As an example, in SF Fig. 2, we present the weighted average BMI levels for the 20-24-year-old age group in future years.

Our study adhered to the Consolidated Health Economic Evaluation Reporting Standards 2022 (CHEERS 2022) statement: updated reporting guidance for health economic evaluations [38].

### Intervention selection

Our selection of interventions for inclusion in this study was drawn from broad strategies identified in a stakeholder engagement process with policy makers in Kenya [26]. We asked the stakeholders to identify existing and new strategies they considered relevant and appropriate for the prevention and control of overweight and obesity in Kenya. From that list of broad strategies, each stakeholder selected their top three for inclusion in our ACE modelling study. We calculated total weighted scores and selected the two highest ranked strategies for inclusion in our study [26]. These were broad strategy scenarios which included: 1) a research-based strategy where the Kenya Agricultural & Livestock Research Organisation (KALRO) promotes and coordinates research to investigate the nutritional value of indigenous foods and 2) a health promotion and education strategy that does not only tell people to eat healthy diets but one that also creates a healthy environment [26]. Under the first strategy scenario, it is envisioned that the research-based evidence would then be used to promote consumption of indigenous foods found to have high nutritional value. For the second strategy scenario, we considered interventions that create a healthy environment as those that seek to reverse the obesogenic nature of environments by influencing environmental factors such as structures, systems, laws, policies, and sociocultural norms [39, 22]. Next, we searched literature to define what the proposed broad strategies might look like in practical terms, describe them as interventions that can be modelled and identify evidence of intervention effectiveness.

### Definition of interventions and evidence of effect

We considered the feasibility of the two broad strategies proposed, timelines required for implementation of specific scenarios/interventions and achievement of effect and, availability of evidence on intervention effect. Because it is hard to fully describe what the broad strategies would look like in future, estimate their implementation costs, and define effect on body mass, we focused on examples of feasible interventions that Kenya could implement as part of initial steps of the broad strategies proposed by stakeholders. We modelled the first broad strategy as two possible intervention scenarios that increased consumption of healthy indigenous foods: change in consumption levels related to supermarket food purchase and change in national consumption levels back to the 1975 average levels of intake (Fig. 1). Under the second broad strategy we selected two policy interventions that Kenya could implement towards creation of healthy food environment: mandatory kilojoule labelling on food served in formal sector restaurants and a tax on sugar-sweetened beverages (SSBs).

Where the effect of an intervention was reported as changes in calories, we multiplied the calories by 4.18 to convert them to kilojoules [42]. We calculated resultant body weight changes using previous work that has determined that in adults, a change in net energy intake of 94 kJ per day (95% CI 88.2 to 99.8) corresponds to 1 kg change in body weight [43–45]. Meaning that in our model, a reduction in daily energy consumption of 94 kJ was assumed to cause 1 kg in weight loss. We then converted weight changes to changes in BMI using the average height by age and sex derived from the Kenya STEPS survey (SF Table 1) [10]. All interventions were modelled as population-based interventions. For comparability, we assumed that the estimated BMI changes resulting from the four interventions were maintained over the lifetime of the modelled population. In Table 1, we describe how each intervention was defined for modelling and the supporting evidence of effect from literature. Additional details of this process are presented in the SF. For each modelled intervention, we present the age and sex specific effect size expressed as a change in BMI units in SF Table 2.

**Table 1:** Summary of how each intervention was modelled and evidence of effect from literature

Evidence from the literature	Definition of modelled scenario	Effect size and time periods over which they take effect
**Broad strategy 1: A research-based strategy that generates evidence that would be used to promote consumption of indigenous foods found to have high nutritional value**		
We searched PubMed and Scopus electronic databases with an aim to identify evidence of the effect of consumption of healthy indigenous foods on either energy intake, body weight or body mass index (SF Table 3). Details of the search process and results are provided in the SF. A total of eight studies were included for full text review [46–53]. Apart from two studies [52, 53], the rest did not provide empirical evidence on the effect size of increased consumption of healthy indigenous foods on either energy intake, body weight or body mass index (SF Table 4). The two studies reported findings from patients who had overweight or obesity. In one study, patients were randomized into 2 groups: group A (n = 142) followed a basic traditional Chinese diet (BTCD), and group B (n = 142) followed a Western standard diet (WSD). After 6 weeks of treatment in the BCTD group, BMI decreased by 0.46 kg/m^2^, versus 0.28 kg/m^2^ in the WSD [53]. In an earlier trail, patients with overweight were divided into two groups: group A undergoing a 1200-kcal basic traditional Chinese diet (BTCD), and group B undergoing a 1200-kcal standard western diet. On 6 weeks after treatment, patients in group A lost more weight (0.37 +/- 0.52) kg than group B (0.26 +/- 0.79) kg [52]. The evidence from the two studies was not used as it reported on patients who had overweight or obesity, rather than the whole population. We built two intervention scenarios based on data on food consumption levels in Sub-Sahara Africa [54] and Kenyan literature on supermarket food purchase and its effect on BMI [40, 55]. More information on this is provided in the SF. For ease in reporting results, we refer to these two intervention scenarios as ‘interventions’.	**First intervention scenario: Change in consumption levels related to supermarket food purchase** We modelled an intervention scenario where we assumed that if people did not purchase foods from supermarket, this would increase consumption of indigenous foods and their BMI would change by -1.8 (SE 0.24) kg/m^2^ [40]. In the cross sectional Kenyan study, the authors found that 53% of their sampled population lived in households that purchased food in supermarkets [40]. We considered the identified studies relevant since supermarket purchase is seen to contribute to a shift from indigenous foods to processed foods and drinks, often provided in larger packaging sizes and accompanied by promotional campaigns [40, 41]. We considered that promotion of consumption of indigenous foods would see people purchase more of these foods instead of the processed foods found in the supermarket.	Effect size= -1.8 (SE 0.24) kg/m^2^ [40]. In absence of data from other sources on number of people in Kenya purchasing foods from supermarkets, we used the 53% estimate from the primary study [40] to scale effect size to reflect the target population for this intervention. Notably, in Kenya, supermarket chains initially set up in the big cities are now expanding to the smaller towns as evidenced by the 3 towns included in this study [40].This has led to urbanization of rural areas and the expansion of the peri-urban territories [56]. Modelled uncertainty distribution: normal The modelled effect size change in BMI is presented in SF Table 2. We model the intervention effect as starting after 5 years (i.e., from year 6 as illustrated in Fig. 1) and increasing linearly to achieve a full effect from the 15th year.
	**Second intervention scenario: Change in national consumption levels back to the 1975 average levels of intake** A scenario where the government funded research on indigenous foods, findings resulted in promotion of the production and consumption of healthy indigenous foods leading to a change in actual food consumption for the population (population reverts to the 1975 levels of food consumption of 2,079 kcal/person/day [54]). The 1975 consumption would be expected to reflect a higher consumption of indigenous foods. This is an idealistic hypothetical scenario with estimated timelines. Model assumptions are explained further in the SF and SF Tables 5 and 6 provide additional data that inform this scenario. This assumption is supported by the recommended energy intake requirements for healthy adults (2,300 kcal/day for men and 1,900 kcal/day for women) [57]. However, since the data available does not report sex specific consumption levels [54], we modelled the reported average consumption levels as applying to both male and female. A return to the 1975 consumption levels in our scenario is on the assumption that only individuals who have overweight and obesity would reduce intake hence not a return to high undernutrition rates (SF Table 6).	In the model, the new consumption levels (1975 levels) are compared to the 2015 levels (taken to represent estimated 2019 levels) [54]. 1975 levels: 2,079 kcal/person/day 2015 levels: 2,360 kcal/person/day Effect size = -281 kcal/person/day. The 281 kcal/person/day reduction in consumption, translates to a shift in the BMI distribution for the population. This is about 1,176 kJ/day, which equates to an average body weight change of 12.5 kg based on Sacks et. al. [43] who estimate that a change in net energy intake of 94 kJ per day (95% CI 88.2 to 99.8) corresponds to a 1 kg change in body weight [21]. The modelled effect size change in BMI is presented in SF Table 2. The resulting BMI change is sex and age specific due to the average height measures by age and sex for the Kenya population (SF Table 1). As we did for the previous scenario, we model the intervention effect as starting from year 6 (Fig. 1) and increasing linearly to achieve a full effect from the 15th year.
**Broad strategy 2: Interventions that Kenya could implement towards creation of healthy food environment**		
**Intervention: Tax on sugar-sweetened beverages (SSBs)** The specification of the tax in this study is similar to that modelled for the Australian population by Veerman et al [59]. Definitions for SSB, ‘own-price’ and ‘cross-price’ elasticities are provided in the SF. The price elasticity data were based on 2015 updated values of a systematic review and meta-analysis of studies in the Unites States of America, Mexico, Brazil, and France [60]. We did not find evidence on cross price elasticity from Kenya or similar setting. However using available evidence, in our sensitivity analysis, we assessed the impact that ‘cross-price elasticities’ (SF Table 7) would have on the outcomes [60].	We assess a 20% valoric tax on SSBs. In the model, the tax leads to higher prices of SSBs and a decrease in the purchases (via price elasticity) and consumption of SSBs and thus a lower total energy consumption. This translates into a reduction in BMI across the Kenyan adult population. We used data from Euromonitor International and Global Dietary Database to derive estimates of current dietary intake data for Kenya (baseline data before tax) (SF Table 8) [61, 62]. The costs per unit price of beverages was sourced from Euromonitor International. SF Table 9 provides the baseline consumption levels with trend applied for 15 years and levels after the intervention in kJ/day/person. Changes in quantity purchased were assumed to lead to changes in what was consumed, with no compensatory changes in physical activity levels.	To estimate how changes in price resulting from the tax would lead to changes in food purchases in the Kenyan adult population, we used global estimates of the ‘own-price’ elasticity for soft drinks (mean − 1.198, 95% CI -1.340 to -1.057) [60]. In the model, we applied a normal distribution to the price elasticity values for uncertainty. We determined estimate changes in mean daily energy intake for each age and sex group based on the estimated changes in purchases and the average energy density of relevant products. The effect size in sex, age specific change in BMI is presented in SF Table 2. Intervention effect starts from 1st year of implementation.
**Intervention: Mandatory kilojoule labelling on food served in formal sector restaurants** We provide a background description of this intervention, and the literature search process in the SF. We identified the latest evidence as a meta-analysis where authors explored the effect of mandatory calorie exposure on both the retailers (41 studies) and consumers (186 studies) [58].	We model an effect of kilojoule labelling on consumer consumption as being 27.21 fewer calories per meal [58].	Effect size = − 27.21 fewer calories per meal. We adjusted the intervention effect modelled to reflect current national estimate of two meals per week consumed outside home [10]. Further, we applied a scaling effect of 0.5 in our model to account for the proportion of food eaten in the formal sector restaurants in Kenya where enforcement is more feasible. Another scaling effect of 0.5 was applied as the evidence of effect is from the US where this regulation is already in place. The propensity of Kenya consumers to select food based on kilojoule information and attitudes towards reading kilojoule labels on menus may differ from that seen in the United States. The effect size in sex, age specific change in BMI is presented in SF Table 2. Intervention effect starts from 1st year of implementation.

CI: Confidence interval, Kcal: Kilocalorie, SE: Standard error

### Estimation of intervention costs

We based our estimates on the World Health Organization (WHO) NCD costing tool country specific estimates for Kenya [63]. This tool uses an ingredients-based (bottom up) approach where all resources required to deliver an intervention are identified, quantified, and a price is assigned to each resource. For the strategy on increased consumption of healthy indigenous foods following investment in research, we identified additional cost estimates from the Kenya National food and nutrition security policy implementation framework 2017–2022 [64]. From the NCD costing, we draw estimates for the cost of the intervention activity ‘Increasing consumption of healthy indigenous foods through marketing’ (SF Table 10). However, we were not able to cost all aspects of our first idealistic intervention. This is because there are many possible approaches to this broad strategy proposed by the stakeholders. For instance, a realistic two-year research plan can include literature analysis of these indigenous foods or food composition analysis to make inferences. Another possibility is implementation of a large research program within a food or agriculture research organisation where costs are a portion of the organisation’s annual budget. This scenario would require a substantial program and sustained effort over time to get the results. It is difficult to know what it would take to get the results or evidence and hence describe exactly what that program would look like. We report our partial costing process and estimates for this broad strategy in SF Table 10 to facilitate further work in this area. The two intervention scenarios under this first broad strategy were excluded from the cost effectiveness analysis, but we calculate the maximum intervention cost that could be incurred whilst ensuring that intervention remains cost-effective and, in the results, we provide this value as a maximum justifiable cost estimate.

For costing the two specific interventions on SSBs tax and menu kilojoule labelling on food served in formal sector restaurants, we followed the costing examples for the six cost components needed for implementation of similar upstream regulatory policy: human resources (for program management, promotion and media advocacy, law enforcement and inspection and national-level technical assistance); training for program staff; meetings involving external agencies; mass media; supplies and equipment [63]. In the absence of a primary costing study, the NCD costing tool provided the best estimates for the items costed under each of the six cost components. Intervention costs were applied for 100 years to reflect the lifetime of the population with a constant implementation cost applied from year three onward (Table 2). We inflated all costs to 2019 (our model base year) based on Kenya consumer price indices [65] and converted the Kenya shillings, to US dollars using the world Bank’s official 2019 exchange rate of 102 [66]. In the model, we applied discounting to future intervention costs for the lifetime period (3% discounting for the base case).

**Table 2:** Costs estimates for the policy interventions included in the cost effectiveness analysis*

Costing aspects	Costing components	Total cost in KShs.	Total cost in USD	Costing time/ period	Cost in KShs. for each year	Cost in USD/year	Source
**Intervention: Tax on sugar-sweetened beverages**							
Tax on sugar-sweetened beverages^#^	Components: Human resources, training, meetings, mass media, supplies & equipment, other	2,945,702,203	28,881,897	100 years			WHO NCD costing tool [63]
				Year 1^a^	91,916,681	901,221	
				Year 2	34,377,747	337,065	
				Year 3-100	28,769,467	282,078	
	***Cost per capita***	**58.65**	**0.58**				
**Intervention: Mandatory kilojoule labelling on food served in formal sector restaurants**							
Mandatory kilojoule labelling on food served in formal sector restaurants^#^	Components: Human resources, training, meetings, mass media, supplies & equipment, other	3,528,254,086	34,593,678	100 years			WHO NCD costing tool [63]
				Year 1^a^	143,510,565	1,407,086	
				Year 2	41,306,290	404,998	
				Year 3-100	34,116,706	334,506	
	***Cost per capita***	**70.25**	**0.69**				

Kshs.: Kenya shillings, USD: United States Dollar

*All costs are inflated to the year 2019. ^#^To estimate the total cost for the intervention per year, we used examples of similar upstream health promoting intervention that we considered require broadly similar implementation resources

Year 1^a^ in our case is 2019, our base case year

### Estimation of healthcare costs

We describe the estimation of the total and disease specific healthcare costs in Kenya in our previous work [67]. In summary, the 2019 total healthcare costs for Kenya were derived from the 2020 WHO Global Health Expenditure Database (SF Table 11) [68]. We apportioned the total healthcare costs across specific sex and age groups based on age specific annual per capita health spending and sex specific spending on healthcare in Kenya [69]. We used data from five studies to calculate estimates of costs per incident or prevalent case of modelled diseases (SF Table 12) [70–74]. Of the specific diseases modelled, we did not identify any literature with costs for eight, i.e. low back pain, osteoarthritis hip, osteoarthritis knee, gout, Alzheimer’s disease and other dementias, cataract, gallbladder and biliary diseases and atrial fibrillation and flutter. For chronic kidney disease, only costs of kidney transplants and dialysis were identified. Using national survey data, we adjusted the disease costs to account for the percentage of people unwell who did not seek care hence did not incur healthcare costs [69].

To derive the yearly costs of all other diseases per person, we subtracted the age and sex specific costs of all modelled diseases from the total health expenditure in the respective sex and age groups (SF Table 11). These costs are included because as preventive interventions prolong life, additional health expenditure is expected in those added years of life [75]. We modelled the healthcare costs in United States Dollar (USD) as extracted from the primary sources and database [68, 70–74]. We attempted to quantify uncertainty by setting a normal distribution for health care costs assuming the standard deviation to be 20% of the point estimates.

### Proportional multi-state lifetable modelling

We developed the *Kenya Obesity Model*, a proportional multi-state lifetable (pMSLT) model [76] to quantify the health and economic impact of changes in prevalence of high BMI from selected preventive interventions for the 2019 Kenya population (50.2 million) (SF Table 13). We modelled the 2019 population from age zero years to avoid the missing cohort effect, meaning that we capture the impact of the interventions on the younger age groups once they are 20 years and more into the future. Details of the *Kenya Obesity Model* are published in our previous works [11, 67]. The pMSLT model has been used in previous evaluation studies to estimate health and economic impacts and cost effectiveness of various obesity-related preventive strategies [20, 59, 77, 78]. The model is divided into a general section, that is a standard cause elimination life table (main lifetable), and a separate section for each disease with an independent illness-death (Markov) process [76]. The model simulates the 2019 Kenya population (≥ 20 years) and estimates intervention related changes in BMI on the incidence of the 37 modelled high BMI related disease types over the lifetime of the population. The modelled diseases are: type 2 diabetes, six cardiovascular conditions (atrial fibrillation and flutter, hypertensive heart disease, ischaemic heart disease, ischaemic stroke, intracerebral haemorrhage, subarachnoid haemorrhage), 18 cancers (breast [female], colon and rectum, gallbladder and biliary tract, kidney, leukaemia [five types], liver [three types], multiple myeloma, oesophageal, ovarian, pancreatic, thyroid, uterine), chronic kidney diseases (four types), four musculoskeletal diseases (gout, low back pain, osteoarthritis hip and osteoarthritis knee), four diseases categorised as ‘other diseases’ for ease in reporting results (Alzheimer’s disease and other dementias, asthma, cataract, gallbladder and biliary diseases).

In comparison with a ‘do nothing’ scenario where the current BMI levels and trend continue unabated for 25 years, reduced incidence of diseases in the intervention population overtime results in reductions in prevalence and mortality (apart from musculoskeletal diseases and cataract that are not linked to disease specific mortality in the model). The changes in incidence after an intervention related change in BMI were calculated using the potential impact fraction (PIF) applying the distribution shift method [79]. The PIF is a measure of effect that calculates the proportional change in average disease incidence (or prevalence or mortality) after a change in the exposure of a related risk factor [79]. The distribution shift method assumes a continuous risk-factor (BMI) distribution (modelled as lognormally distributed [11]) with continuous relative risks (RRs) from GBD 2019 [80] modelled as normally distributed [81]. The RR estimates (mean, lower and upper levels) by age and sex are the relative risk of morbidity (incidence in our model) from a high-BMI-related disease, per 5 BMI-unit (5 kg/m^2^) increase. We consider the distribution of BMI within each age-sex category and only model benefits for the part of the distribution above BMI 22·5 kg/m^2^. In the PIF calculation, we used 22·5 kg/m^2^ as our lower boundary BMI. This is in line with the available evidence which suggests that the risk of NCDs that are commonly seen in adults starts rising from around BMI 22·5 kg/m^2^ [80]. We applied the following PIF equation:

$$PIF = \frac{{\int\limits_a^b {RR(x)P(x)dx\, - \int\limits_a^b {RR(x){P^*}(x)dx} } \,}}{{\int\limits_a^b {RR(x)P(x)dx} }}$$

x: risk factor level, RR(x): relative risk function, P(x): original risk factor distribution, P*(x): intervention risk factor distribution, a,b: the integration boundaries, dx: integration with respect to x

The changes in incidence of the disease subsequently lead to changes in morbidity and mortality rates. Changes in disease-related quality of life at every age were calculated using disease-specific disability weights [2, 11, 82]. The new disease specific mortality and morbidity rates and changes in costs from the disease sections are fed into a life table to calculate the number of HALYs and total cost savings. Changes in healthcare expenditure were estimated both for modelled high BMI related NCDs and overall healthcare costs in added years of life [75]. A detailed report on the sources of data, preparation of data and estimates of the epidemiological input data used in the model is provided elsewhere in our previous work [11].

### Cost-effectiveness modelling

Considering a health system perspective, we conducted a cost effectiveness analysis for two interventions, the 20% SSB tax and mandatory kilojoule menu labelling. We calculated the incremental cost-effectiveness ratios (ICERs), defined as the difference in net costs of the intervention compared to no intervention comparator, divided by the difference in HALYs. Net costs included intervention costs and healthcare costs (including costs in added life years). We assumed all interventions to be fully implemented and running at their full effectiveness potential. We assumed that the impact of interventions on BMI (intervention effect) were maintained over the lifetime of the modelled population.

Given the absence of cost effectiveness thresholds in Kenya, we used the WHO benchmarks for definition of cost-effectiveness for each modelled intervention. We defined a very cost-effective intervention as ICER < 1,801 USD (i.e., the gross domestic product [GDP] per capita for Kenya in 2019) [83] per HALY gained, and a cost-effective intervention being defined as ICER < 5,403 USD (i.e., 3 times the Kenya GDP per capita) per HALY gained. Cost-saving was defined as having a negative net cost.

#### Other outcomes reported

We computed the tax revenue that would be generated following implementation of the SSB tax. The assessment of productivity gains was also included as this was already built into the model in our previous work [67]. Productivity gains were not part of the cost effectiveness analysis. We used the Human Capital Approach [24] to estimate productivity gains resulting from a reduction in high BMI -related 1) mortality, 2) mortality and morbidity (combined) and 3) morbidity. Further details on this are provided in the SF. Additionally, we included net monetary benefit accruing from the two interventions assessed at this stage. This was calculated as the total healthcare cost savings and productivity gains realised from each intervention less total intervention cost. The productivity gain estimate used in the net monetary benefit calculation is the obesity-related mortality and morbidity (combined) outcome [67]. We also report the net monetary benefit without inclusion of productivity gains. In the main analysis, we applied a discount rate of 3% to costs and HALYs as recommended in Drummond et al [24]. We shared details on costing process, cost estimates, cost effectiveness threshold for Kenya with the stakeholders for their review and comment.

### Uncertainty and sensitivity analysis

While incorporating uncertainty from model inputs, we conducted a probabilistic sensitivity analysis using Monte Carlo simulation with bootstrapping (2000 iterations) to estimate the 95% uncertainty intervals around BMI, HALYs and cost outcomes in the base case scenarios.

This was implemented using Ersatz version 1.35 software [84]. The point estimate and 95% uncertainty intervals (UI) were defined as the 50th and 2.5th -97.5th percentiles, respectively.

We conducted univariate sensitivity analyses to explore the impact of variation in discount rates (0% and 5%). For the SSB tax intervention, additional sensitivity scenarios were included by varying the tax from 20% (base case) to 10% and 30%, varying the pass on rate from 100% (base case) to 80% and 120% and, by applying cross price elasticity to the base case scenario. In order to compare like for like in the sensitivity analysis we run all scenarios with uncertainty off.

Ethics approval was not required for this analysis. However, this study was carried out as part of a larger study that has ethical approval from the Griffith University Human Research Ethics Committee (GU Ref No: 2019/707) and the Kenyatta National Hospital/University of Nairobi *Ethics* & Research Committee (P81/02/2021).

## Results

### Reduction in overweight and obesity

If implemented in 2019, a 20% tax on sugar sweetened beverages could result in a reduction of ~ 44,232 people with overweight (Males = 28,902 UIs 22,229 to 37,442, Females = 15,330 UIs 11,800 to 20,052) and ~ 32,140 people with obesity (Males = 10,090 UIs 7,820 to 12,802, Females = 22,050 UIs 17,504 to 27,497) (Fig. 2). The mandatory kilojoule menu labelling could result in a reduction of ~ 33,691 people with overweight (males = 20,665 UIs 7,220 to 34,126, Females = 13,026 UIs 4,531 to 21,578) and ~ 27,076 people with obesity (Males = 7,579 UIs 2,655 to 12,492, Female = 19497 UIs 6,817 to 32,180) while the change in consumption levels related to supermarket food purchase intervention could result in a reduction of ~ 1.1 million people with overweight (Males = 634,968 UIs 474,251 to 788,201, Females = 416,069 UIs 296,171 to 538,257) and ~ 731,268 people with obesity (Males = 210,734 UIs 161,637 to 255,861, Females = 520,534 UIs 389,434 to 645,705). The change in national consumption levels back to the 1975 average levels of energy intake could lead to the highest gains with a reduction of over 4.3 million people with overweight (Male = 1,955,479 UIs 1,900,480 to 2,009,639, Female = 2,378,665 UIs 2,250,201 to 2,510,957) and over 2.3 million with obesity (Males = 534,684 UIs 524,115 to 544,838, Females = 1,771,755 UIs 1,722,546 to 1,820,027). Apart from the supermarket food purchase scenario where the evidence of effect was a change in BMI, the intervention effect was modelled at the level of change in consumption.

### Changes in disease incidence, prevalence, and mortality

Over the first 25 years, implementation of a 20% tax on sugar sweetened beverages could prevent an estimated total of 80,060 incident cases of diseases across the diseases modelled. The results tables indicate the uncertainty intervals for all estimates reported. Over the same period, a total of 5,083 deaths for type 2 diabetes (T2DM), cardiovascular diseases, chronic kidney disease (CKD), cancers, Alzheimer’s disease and other dementias, asthma, gallbladder and biliary diseases could be avoided (Table 3). The greatest impact of averted new disease cases was seen in T2DM. In the 25th year (the year 2044), across all modelled diseases, ~ 42,511 prevalent cases of disease could be avoided.

The mandatory kilojoule menu labelling intervention could see a total of 57,319 incident cases of diseases avoided over the first 25 years. In the same period, a total of 4,157 deaths avoided for T2DM, cardiovascular diseases, CKD, cancers, Alzheimer’s disease and other dementias, asthma, gallbladder and biliary diseases (Table 3). In the 25th year, ~ 28,389 prevalent cases of disease could be avoided. The greatest impact of averted new diseases cases was seen in musculoskeletal diseases followed by T2DM, cardiovascular conditions and CKDs (Table 3). For the other two interventions where a change in national consumption levels back to the 1975 average levels of energy intake and in consumption levels related to supermarket food purchase were modelled, the greatest reductions of new disease cases were also seen in musculoskeletal diseases followed by T2DM, cardiovascular conditions and CKDs.

For the change in consumption levels related to supermarket food purchase, over the first 25 years, a total of 1.2 million incident cases of diseases could be averted and a total of 72,581 deaths avoided for T2DM, cardiovascular diseases, CKDs, cancers, Alzheimer’s disease and other dementias, asthma, gallbladder and biliary diseases. In the 25th year (year 2044), ~ 699,764 prevalent cases of modelled diseases could be avoided (Table 3). A change in national consumption levels back to the 1975 average levels of energy intake yielded the largest magnitude of impact. Over 4.3 million incident cases of diseases could be averted, and 247,594 deaths avoided over the first 25 years (Table 3). In the year 2044, ~ 2.4 million prevalent cases of disease could be avoided. For each intervention, changes by disease are reported in SF Tables 14 to 17.

**Table 3:** Projected number of incident cases, prevalent cases and avoided deaths by disease group

Variable	Cumulative incident cases averted (2019 to 2044)	Prevalent cases avoided (for the year 2044)	Avoided deaths by disease group^#^ (2019 to 2044)
	**Total, n (95% UI)**	**Total, n (95% UI)**	**Total, n (95% UI)**
Intervention/ Disease group			
**20% SSB tax**			
T2DM	29,731	20,624	1,122
	(21,558 − 39,552)	(15,122 − 27,281)	(832–1,460)
Cardiovascular diseases	12,319	6,802	3,270
	(9,629 − 15,497)	(5,322–8,578)	(2,443–4,269)
Musculoskeletal diseases	22,239	6,589	N/A
	(17,483 − 27,793)	(4,759–8,862)	N/A
CKD	7,895	5,436	246
	(3,970 − 13,694)	(2,457–9,920)	(138–390)
Cancers	375	52	275
	(231–567)	(34–75)	(155–436)
Other diseases	7,500	3,008	169
	(5,482–9,792)	(2,108–4,055)	(121–224)
**Mandatory kj menu labelling**			
T2DM	16,696	11,366	667
	(5,692–29,220)	(3,894–19,699)	(230–1,150)
Cardiovascular diseases	10,087	5,188	2,835
	(3,636–17,138)	(1,859–8,755)	(1,032–4,825)
Musculoskeletal diseases	17,305	5,038	N/A
	(6,121–29,131)	(1,706–8,967)	N/A
CKD	6,911	4,509	240
	(1,930–14,533)	(1,151–9,911)	(71–473)
Cancers	346	44	255
	(117–646)	( 15–80)	(81–491)
Other diseases	5,974	2,243	160
	(2,145–10,271)	(769–3,962)	(58–279)
Change in consumption levels related to supermarket food purchase*			
T2DM	350,442	259,069	9,788
	(239,273–473,272)	(176,932–348,568)	(6,671–13,245)
Cardiovascular diseases	220,539	132,044	50,329
	(154,010–298,034)	(93,265–174,789)	(33,923–71,039)
Musculoskeletal diseases	388,189	139,926	N/A
	(275,259–508,823)	(93,844–192,909)	N/A
CKD	147,041	106,290	4,026
	(71,384–264,816)	(48,098–199,049)	(2,094–6,397)
Cancers	7,865	1,353	5,590
	(4,545–12,278)	(850–1,998)	(2,966–9,112)
Other diseases	128,707	61,082	2,847
	(89,927–172,749)	(41,660–83,966)	(1,956–3,875)
Change in national consumption levels back to the 1975 average levels of energy intake *			
T2DM	1,042,887	773,500	29,798
	(827,863–1,264,903)	(620,520–931,184)	(23,738–36,059)
Cardiovascular diseases	757,453	454,457	172,514
	(636,593–886,850)	(389,766–523,037)	(136,188–216,066)
Musculoskeletal diseases	1,494,251	544,707	N/A
	(1,248,593–1,762,481)	(419,577–682,021)	N/A
CKD	519,329	379,280	13,375
	(264,520–845,809)	(179,833–641,140)	(7,748–19,961)
Cancers	30,191	5,330	21,326
	(19,099–44,144)	(3,608–7,337)	(12,071–32,777)
Other diseases	486,004	236,005	10,580
	(394,749–590,463)	(185,476–293,871)	(8,423–12,986)

Total refers to estimates for both male and female. BMI: body mass index, kJ: kilojoule, n (95% UI): mean (95% Uncertainty interval), N/A: not applicable, SSB: sugar sweetened beverages

^#^Musculoskeletal diseases and cataract are not linked to disease specific mortality

*For the modelled scenarios that could lead to an increased consumption of healthy indigenous foods (Interventions: a change in national consumption levels back to the 1975 average levels of energy intake and a change in consumption levels related to supermarket food purchase), the intervention effect in the model starts from year 6 (after 5 years of envisioned research) and increases linearly to achieve a full effect from the 15th year

T2DM: Diabetes mellitus type 2, cardiovascular diseases include ischemic heart disease, ischemic stroke, intracerebral haemorrhage, subarachnoid haemorrhage, hypertensive heart disease, atrial fibrillation and flutter

Musculoskeletal diseases include osteoarthritis hip, osteoarthritis knee, low back pain and gout

Chronic kidney disease (CKD) includes CKD due to diabetes mellitus type 2, CKD due to glomerulonephritis, CKD due to hypertension and CKD due to other and unspecified causes

Cancers include oesophageal cancer, colon cancer, liver cancer due to alcohol use, liver cancer due to hepatitis B, liver cancer due to hepatitis C, gallbladder and biliary cancer, pancreatic cancer, breast cancer, uterine cancer, ovarian cancer, kidney cancer, thyroid cancer, acute lymphoid leukaemia, acute myeloid leukaemia, chronic lymphoid leukaemia, chronic myeloid leukaemia, other leukaemia, and multiple myeloma

*Other diseases* include gallbladder and biliary diseases, asthma, Alzheimer’s disease and other dementias and cataract

### Changes in health adjusted life years, life years and overall deaths

Over the lifetime of the 2019 population, the impact of the 20% tax on sugar sweetened beverages could translate to ~ 203,266 HALYs, ~ 151,718 HALYs for the mandatory kilojoule menu labelling, over 3.6 million HALYs for the change in consumption levels related to supermarket food purchase intervention and over 13 million for the change in national consumption levels back to the 1975 average levels of energy intake (Table 4). In the year 2044, over 3,000 deaths could be avoided following the implementation of a 20% tax on sugar sweetened beverages. For the same period, implementation of the mandatory kilojoule menu labelling could see over 2,000 deaths avoided, a change in consumption levels related to supermarket food purchase could lead to over 53,000 deaths avoided while a change in national consumption levels back to the 1975 average levels of energy intake could see ~ 185,869 deaths avoided.

**Table 4:** Effects of each intervention on HALYs, LYs and overall deaths

Intervention	20% SSB tax	Mandatory kJ menu labelling	Change in consumption levels related to supermarket food purchase	Change in national consumption levels back to the 1975 average levels of energy intake
Variable	**Total, mean**	**Total, mean**	**Total, mean**	**Total, mean**
	**(95% UI)**	**(95% UI)**	**(95% UI)**	**(95% UI)**
HALYs gained over the lifetime	203,266	151,718	3,677,675	13,108,472
	(163,752–249,621)	(55,257–250,412)	(2,661,365–4,789,915)	(11,404,317–15,152,341)
LYs gained over the lifetime	124,970	104,945	2,569,421	9,356,747
	(95,712–160,157)	(37,962–177,605)	(1,785,831–3,533,570)	(7,670,302–11,471,801)
HALYs gained by the year 2030	7,134	5,761	14,681	48,770
	(5,810–8,628)	(2,071–9,441)	(10,854–18,507)	(44,646–53,186)
HALYs gained by the year 2044	39,424	29,756	417,782	1,403,031
	(31,942–47,744)	(10,643–48,531)	(307,424–531,865)	(1,264,258–1,556,872)
Deaths* avoided by the year 2030	623	608	2,175	7,362
	(484–782)	(217–1,009)	(1,509–2,937)	(6,129–8,817)
Deaths avoided by the year 2044	3,246	2,744	53,709	185,869
	(2,506–4,118)	(981–4,594)	(37,558–72,694)	(155,703–222,881)

Total refers to estimates for both male and female. HALYs: health adjusted life years, kJ: kilojoule, LYs: life years, UI: Uncertainty interval, SSB: sugar sweetened beverages

We modelled the entire 2019 population in Kenya with risks rising only from age 20 and no burden among children

*This is the overall mortality number from lifetable where we count both the reduction in mortality from BMI-related diseases and the increase in mortality due to other causes

### Economic impact and cost effectiveness

Over the lifetime of the 2019 population, the 20% SSB tax could lead to a reduction in healthcare cost by about USD 140 million and the mandatory kilojoule menu labelling could result to healthcare cost savings of USD 83 million (Table 5). A change in consumption levels related to supermarket food purchase could yield over USD 1.9 billion in healthcare cost savings and a change in national consumption levels back to the 1975 average levels of energy intake could yield over USD 6.2 billion (Table 5). Each intervention resulted in large productivity gains. Over the lifetime, productivity gains due to obesity-related mortality and morbidity (combined) were in excess of USD 1.8 billion for the 20% tax on sugar sweetened beverages, over USD 1.2 billion for the mandatory kilojoule menu labelling, over USD 27 billion for the intervention on a change in consumption levels related to supermarket food purchase and, over USD 92 billion for the change in national consumption levels intervention.

The intervention cost (discounted) for the 20% tax on sugar sweetened beverages was estimated to cost USD 9.9 million while the mandatory kilojoule menu labelling would cost USD 12.0 million (Table 5). These two interventions were found to be very cost effective when cost offsets were not included: USD 49 per HALY gained (95% UI 39–60) for the 20% tax on sugar sweetened beverages and USD 92 per HALY gained (95% UI 47–202) for the mandatory kilojoule menu labelling. When cost offsets were included the two interventions were dominant (health promoting and cost-saving) (Table 5). For 20% tax on sugar sweetened beverages, the net monetary benefit without productivity was ~ USD 0.13 billion and ~ USD 2 billion with productivity. The mandatory kilojoule menu labelling resulted to a net monetary benefit of ~ USD 0.07 billion without productivity and ~ USD 1.3 billion with productivity. In addition to the above economic gains, the 20% tax on sugar sweetened beverages would also raise USD 71 billion in revenue for the government or USD 98 billion without discounting (Table 5).

**Table 5:** The economic impact and cost effectiveness

Intervention	20% SSB Tax	Mandatory kJ menu labelling	Change in consumption levels related to supermarket food purchase	Change in national consumption levels back to the 1975 average levels of energy intake
Variable	**Total, mean**	**Total, mean**	**Total, mean**	**Total, mean**
	**(95% UI)**	**(95% UI)**	**(95% UI)**	**(95% UI)**
**Healthcare cost savings, productivity gains, intervention cost (in Million USD)**				
Healthcare cost savings over the lifetime	140	83	1,925	6,226
	(96–188)	(29–152)	(1,152–2,780)	(3,832–8,428)
Healthcare cost savings by the year 2044	58	39	661	2,150
	(45–74)	(14–67)	(473–856)	(1,830–2,495)
Productivity gains (morbidity) by year 2044	441	290	4,443	14,472
	(350–547)	(104–482)	(3,255–5,654)	(12,906 − 16,111)
Productivity gains (mortality) by year 2044	159	122	1,514	4,965
	(119–207)	(44–208)	(1,067 − 2,017)	(4,163–5,832)
Productivity gains (mortality & morbidity) by year 2044	535	361	5,336	17,400
	(419–664)	(130–602)	(3,901–6,814)	(15,395 − 19,506)
Productivity gains (morbidity) over the lifetime	1,413	896	20,860	68,877
	(1,110–1,761)	(321–1,495)	(15,206 − 26,705)	(60,806 − 77,5957)
Productivity gains (mortality) over the lifetime	724	519	12,045	39,981
	(528–969)	(190–902)	(8,294 − 16,485)	(32,628 − 48,601)
Productivity gains (mortality & morbidity) over the lifetime	1,841	1,202	27,961	92,453
	(1,424–2,309)	(429–2,020)	(20,216 − 36,196)	(79,619 − 105,929)
Intervention cost (discounted)	9.9	12.0		
Intervention cost (undiscounted)	28.9	34.6		
**ICER (USD per HALY)**				
ICER (without cost offsets)*	49	92		
	(39–60)	(47–202)		
ICER (with cost offsets)*	‘dominant’	‘dominant’		
	‘dominant’	‘dominant’		
**Net monetary benefit and tax revenue in Billion USD**				
Net monetary benefit with productivity	2.0	1.3		
	(1.5–2.5)	(0.5–2.1)		
Net monetary benefit without productivity	0.13	0.07		
	(0.09–0.18)	(0.02–0.14)		
Tax revenue by the year 2044 (discounted)	71			
	(69–73)			
Tax revenue by the year 2044 (undiscounted)	98			
	(95–101)			

Productivity gains included in the net monetary benefit calculation was the obesity-related mortality and morbidity (combined) outcome

Total refers to estimates for both male and female. kJ: kilojoule, SSB: sugar sweetened beverages, UI: Uncertainty interval, USD: US dollars. ICER: incremental cost-effectiveness ratios, dominant (dominant: health promoting and cost-saving).

The following modelled diseases were not costed: low back pain, osteoarthritis hip, osteoarthritis knee, gout, Alzheimer’s disease and other dementias, cataract, gallbladder and biliary diseases and atrial fibrillation and flutter (AFF). We considered that costs for AFF may already be included under the other cardiac related conditions costed.

Productivity gains estimates: gains resulting from a reduction in obesity-related morbidity, mortality and obesity-related mortality and morbidity (combined).

We modelled the entire 2019 population in Kenya with risks rising only from age 20 and no burden among children.

*cost offsets are healthcare cost savings. Net monetary benefit was calculated as the total healthcare cost savings and productivity gains realised less total intervention cost. 

In Table 6, we calculate the maximum justifiable cost for a research-based strategy that leads to increased production and consumption of healthy indigenous foods. This is the maximum intervention cost that could be incurred whilst ensuring that intervention remains cost-effective or very cost effective.

If healthcare cost savings alone are considered and used to guide on the maximum intervention cost, for the first intervention scenario where there is a change in consumption levels related to supermarket food purchase, the maximum intervention cost that could be incurred whilst ensuring that intervention remains cost-effective is USD 1,924,647,709, for the second intervention scenario on a change in national consumption levels back to the 1975 average levels of intake, maximum justifiable would be USD 6,225,633,667. If considering both the healthcare cost savings and the value of HALYs gained, how much one is willing to pay for a HALY would determine the maximum justifiable cost. Where a threshold of USD 5,403 is applied as value per HALY gained (i.e., 3 times the GDP per capita for Kenya in 2019) [11], for the first intervention scenario, the maximum justifiable cost would be USD 21,795,123,562 while the second intervention scenario would be USD 77,050,705,862. Where a threshold of USD 1,801 is applied as value per HALY gained (i.e., the GDP per capita for Kenya in 2019, for the first intervention scenario, the maximum justifiable cost would be USD 8,548,139,660 while the second intervention scenario would be USD 29,833,991,065.

**Table 6:** The maximum justifiable cost

Intervention: A research-based strategy that leads to increased production and consumption of healthy indigenous foods			
	Maximum justifiable cost when healthcare cost savings alone are considered (USD)	Maximum justifiable cost when HALYs gained, and healthcare cost savings are considered (USD)	
		Value of HALYs being USD 1,801* (very cost-effective threshold)	Value of HALYs being USD 5,403* (cost effective threshold)
1. When the intervention is modelled as a change in consumption levels related to supermarket food purchase	1,924,647,709	8,548,139,660	21,795,123,562
2. When the intervention is modelled as a change in national consumption levels back to the 1975 average levels of intake	6,225,633,667	29,833,991,065	77,050,705,862

*The value is calculated by multiplying the HALYs gained with 5,403 USD (i.e., 3 times the GDP per capita for Kenya in 2019, the definition of a cost-effective intervention applied in our study) or with 1,801 (the GDP per capita for Kenya in 2019, the definition of a very cost-effective intervention applied in our study). These outputs are from the main analysis (base case) where we applied a discount rate of 3% to costs and HALYs

### Sensitivity analysis

When 5% discounting was applied, the lifetime HALYs gained reduced by ~ 50% for all interventions when compared to base case (3% discount rate). When no discounting was applied the HALYs gained over the lifetime were about 4 times the base case scenario (Table 7). When 5% discount rate was applied, the lifetime healthcare cost savings reduced by about 30% compared to base case. The lifetime healthcare cost savings were 2 times those in base case when 0% discount rate was applied. As expected, compared to base case scenario, the health and economic gains increased when a 30% SSB tax was implemented and reduced when a 10% SSB tax was implemented or when cross price elasticities were applied. Including a 120% pass on rate increased both the lifetime HALYs gained and healthcare cost savings by 16% while an 80% pass on rate reduced the lifetime HALYs gained and healthcare cost savings by 17% (compared to base case) (Table 7). In the sensitivity analysis, the two interventions assessed for cost effectiveness were found to be very cost effective (without cost offsets) and dominant (with cost offsets included).

**Table 7:** Health and economic impact for various scenarios included in sensitivity analysis

Intervention	20% SSB Tax				20% SSB Tax base case setting but		10% SSB Tax	30% SSB Tax	
Sensitivity scenario	**0% discount rate**	**3% discount (base case)**	**5% discount rate**	**3% discount with CPE applied**	**with 120% pass on rate**	**with 80% pass on rate**	**3% discount rate**	**3% discount rate**	
**Variable**	**Point estimates for both male and female combined**								
HALYs gained over the lifetime of the 2019 population	752,193	198,471	96,204	95,485	229,632	164,888	109,363	272,362	
HALYs gained by the year 2044	64,025	38,905	28,580	18,320	45,013	32,324	21,446	53,380	
**Healthcare cost savings, productivity gains, intervention cost (in Million USD)**									
Healthcare cost savings over the lifetime	300	141	86	75	163	117	78	193	
Healthcare cost savings by the year 2044	92	57	43	28	66	48	32	79	
Productivity gains (morbidity) by year 2044	709	437	325	216	506	363	241	600	
Productivity gains (mortality) by year 2044	260	155	112	64	179	129	85	212	
Productivity gains (mortality & morbidity) by year 2044	861	528	390	253	611	439	291	724	
Productivity gains (morbidity) over the lifetime	3,880	1,396	784	716	1,615	1,160	770	1,915	
Productivity gains (mortality) over the lifetime	2,139	697	364	304	806	579	384	956	
Productivity gains (mortality & morbidity) over the lifetime	5,129	1,803	996	894	2,086	1,498	994	2,473	
Intervention cost (discounted)	28.9	9.9	6.6	9.9	9.9	9.9	9.9	9.9	
**ICER (USD per HALY)**									
ICER (without cost offsets)*	38	50	68	103	43	60	90	36	
ICER (with cost offsets)*	‘dominant’	‘dominant’	‘dominant’	‘dominant’	‘dominant’	‘dominant’	‘dominant’	‘dominant’	
**Net monetary benefit and tax revenue in Billion USD**									
Net monetary benefit with productivity	5.40	1.93	1.08	0.96	2.24	1.61	1.06	2.66	
Net monetary benefit without productivity	0.27	0.13	0.08	0.06	0.15	0.11	0.07	0.18	
Tax revenue by the year 2044 (discounted)	98.02	71.13	59.10	71.13	68.39	74.08	39.47	96.94	
Tax revenue by the year 2044 (undiscounted)	98.02	98.02	98.02	98.02	94.24	102.08	54.39	133.58	
**Intervention**	**Mandatory kJ menu labelling**			**Change in consumption levels related to supermarket food purchase**			**Change in national consumption levels back to the 1975 average levels of energy intake**		
**Sensitivity scenarios with varied discount rates**	**0%**	**3%**	**5%**	**0%**	**3%**	**5%**	**0%**	**3%**	**5%**
**Variable**	**Point estimates for both male and female combined**								
HALYs gained over the lifetime of the 2019 population	579,204	149,672	72,157	14,968,297	3,568,793	1,590,360	55,075,938	12,829,960	5,644,586
HALYs gained by the year 2044	48,494	29,658	21,888	724,078	409,911	285,225	2,453,514	1,387,948	965,274
**Healthcare cost savings, productivity gains, intervention cost (in Million USD)**									
Healthcare cost savings over the lifetime	145	85	55	3,601	1,945	1,166	10,579	6,308	3,830
Healthcare cost savings by the year 2044	62	39	30	1,126	652	461	3,694	2,137	1,509
Productivity gains (morbidity) by year 2044	467	291	217	7,684	4,394	3,079	25,214	14,417	10,101
Productivity gains (mortality) by year 2044	202	121	88	2,654	1,477	1,015	8,807	4,900	3,366
Productivity gains (mortality & morbidity) by year 2044	585	362	269	9,234	5,257	3,672	30,358	17,279	12,067
Productivity gains (morbidity) over the lifetime	2,474	895	506	61,790	20,561	10,757	206,625	68,447	35,712
Productivity gains (mortality) over the lifetime	1,553	510	269	38,478	11,626	5,679	129,953	39,130	19,072
Productivity gains (mortality & morbidity) over the lifetime	3,381	1,193	663	84,260	27,350	14,073	282,515	91,298	46,850
Intervention cost (discounted)	34.6	12.0	8.1						
**ICER (USD per HALY)**									
ICER (without cost offsets)*	60	80	112						
ICER (with cost offsets)*	‘dominant’	‘dominant’	‘dominant’						
**Net monetary benefit in Billion USD**									
Net monetary benefit with productivity	3.49	1.27	0.71						
Net monetary benefit without productivity	0.11	0.07	0.05						

HALYs: health adjusted life years, kJ: kilojoule, SSB: sugar sweetened beverages, USD: US dollars. ICER: incremental cost-effectiveness ratios, dominant (health promoting and cost-saving). The following modelled diseases were not costed: low back pain, osteoarthritis hip, osteoarthritis knee, gout, Alzheimer’s disease and other dementias, cataract, gallbladder and biliary diseases and atrial fibrillation and flutter (AFF). Productivity gains estimates: gains resulting from a reduction in obesity-related morbidity, mortality and obesity-related mortality and morbidity (combined). *cost offsets are healthcare cost savings. Net monetary benefit was calculated as the total healthcare cost savings and productivity gains realised less total intervention cost. Productivity gains included in the net monetary benefit calculation was the obesity-related mortality and morbidity (combined) outcome. We modelled the entire 2019 population in Kenya with risks rising only from age 20 and no burden among children. All sensitivity scenarios run with uncertainty off

## Discussion

### Statement of principal findings

Our aim was to assess the impact of the different proposed interventions; taxation on sugar sweetened beverage, mandatory kilojoule menu labelling on food served in formal sector restaurants, change in consumption levels related to supermarket food purchase and change in national consumption levels (return to the 1975 average levels of intake) in control of obesity and overweight in Kenya.

Our findings show that all interventions evaluated would yield substantive reduction in people with overweight and obesity which translated to significant health gains, healthcare cost savings and productivity gains. Over the lifetime of the 2019 population, the impact on HALYs translated to 4, 3, 73 and 261 HALYs per 1,000 persons for a 20% tax on sugar-sweetened beverages, mandatory kilojoule menu labelling, a change in consumption levels related to supermarket food purchase patterns and for a change in national consumption back to the 1975 average levels of energy intake, respectively. There were wide differences in the sizes of the impacts with the two specific interventions (a 20% tax on sugar-sweetened beverages and mandatory kilojoule menu labelling) resulting in good impact while the two based on more broad scenarios that were assumed to result in large changes in consumption patterns, had very large impacts.

For context, from the change in national consumption levels back to the 1975 average levels of intake, the lifetime healthcare cost savings are 1.4 times the 2019 Kenya total healthcare expenditure or 6.2% of the 2019 GDP. Savings from a change in consumption levels related to supermarket food purchase translate to 42% of the 2019 Kenya total healthcare expenditure or 2% of GDP, savings from the 20% tax on sugar sweetened beverages were 3% of total healthcare expenditure or 0.1% of GDP while those from the mandatory kilojoule menu labelling translated to 2% of total healthcare expenditure or 0.08% of GDP. The cost savings realised will not only benefit the government but will also bring relief to many households due to the current high out-of-pocket healthcare costs incurred by individuals in Kenya (~ 28%) [85]. For each intervention, the lifetime productivity gains due to obesity related morbidity and mortality (combined) were about 14 times greater than the corresponding healthcare costs savings.

The intervention costs were 3% and 6% of the projected healthcare cost savings. Translating to about 0.1% of the 2019 total healthcare expenditure in Kenya for both interventions. This is indicative of the feasibility of the two specific interventions in Kenya. The two specific interventions were assessed for cost effectiveness and found to be very cost effective (without cost offsets) and dominant (health promoting and cost-saving) with cost offsets included, i.e., from a health sector perspective.

When various sensitivity scenarios were assessed, both interventions were still found to be very cost effective (without cost offsets) and dominant (with cost offsets included).

### Comparison with other studies

To our knowledge, this is the first study to assess the potential economic and health impact and cost effectiveness of interventions for the prevention and control overweight and obesity in adults in Kenya. A similar study has been done by Ananthapavan et al. who assessed the cost effectiveness of obesity prevention policies in Australia [20]. The authors used similar modelling methods. Of the 16 interventions that they assessed, they included mandatory kilojoule labelling on fast food and a 20% sales tax on SSB. They described the kilojoule labelling on fast food as mandatory legislation for fast food outlets displaying energy content of foods and drinks on menus accompanied by a government education campaign. Both interventions yielded health gains and were cost saving. In the assessment for cost effectiveness, the two interventions were found to be dominant (health promoting and cost-saving). The menu kilojoule labelling legislation was found to have high feasibility, sustainability and acceptability by public and government [20]. Other modelling studies carried out in high income countries have also found that a tax on SSBs resulted to changes in average body mass translating to substantial health gains, healthcare cost savings [59, 86], and the tax intervention was found cost effective [87, 88]. Similar studies have evaluated the health and/or economic impact of SSB tax in low-, middle- and upper-middle income countries [89–96]. Some differences in these studies include the varied tax types and thresholds included in the studies, sources of evidence of effect, model decisions/assumptions made, stratification of analyses e.g., by whole population or across income groups and, different health or economic outcomes are assessed. However, across all the studies, a tax on SSBs was found to reduce obesity related morbidity and premature deaths, reduce healthcare costs and to be cost effective.

### Strengths and limitations

We apply established modelling methods to assessing the health and economic impact and cost effectiveness of interventions for the prevention and control of overweight and obesity in Kenya [11, 59, 76]. As a strength, our model includes a comprehensive list of high BMI related NCDs as identified in the literature. In the base case analysis, we included extensive uncertainty to account for uncertainty in data and evidence base. We also test some sensitivity scenarios to assess robustness of our study results. In addition, we expanded our study to include the assessment of productivity gains, net monetary benefit and estimation of the revenues that taxation could generate for the government.

Another key strength of our study is that the selection of our interventions was informed by stakeholders from various sectors across government, development agencies, higher education and research and civil society who influence priorities for NCD control in Kenya [26]. Additionally, the model scenarios and evidence of effect for each intervention were underpinned by evidence in literature [40, 54, 55, 58, 60].

Due to limitations of available evidence, the likely impact of kilojoule intake on body weight was based on the assumption that there is no compensatory behaviour (e.g., physical inactivity following reduced consumption). Further research on possibility or extent of compensatory behaviour could enhance the evidence base.

Though all our interventions targeted whole populations, we used available evidence to scale the reduction in BMI associated with two interventions: not purchasing foods from supermarket and with mandatory kilojoule labelling on food served in formal sector restaurants [10, 40]. To estimate the population purchasing foods from supermarkets, we used findings from Demmler et al, who highlighted that their study sample (n = 550) was not representative of the whole country [40]. Nationally representative data on the number of people who make purchases in supermarkets could inform the size of the target population for this intervention in Kenya.

Enforcement of the mandatory kilojoule labelling is more likely in the formal food retail outfits as opposed to the informal sector. Since there is a large informal food market in Kenya, data on estimates of formal verses informal sector could help tailor the intervention further to the Kenya context. Nonetheless, it is important to note that there is a rapid growth of formal sector restaurants, especially fast-food retailers in Kenya particularly in the urban sector where currently most of the overweight and obesity burden is found (25% of those residing in urban areas have overweight compared to 16% in rural areas) [10]. Additionally, urbanisation is spreading rapidly within the rural areas of the country and a rise in BMI is expected there too [56].

The data on disease costs were specific to Kenya and similar settings and estimates for total healthcare expenditure and intervention costs were country specific. However, as detailed in our previous work [67], we encountered some limitations in costing. In summary, our disease cost estimates may be high since identified costing studies were hospital based which reflect cost of treatment for advanced disease cases. We used disease input data from GBD that adopts broader case definitions where prevalent numbers for instance, may include people not aware they have the condition. To limit the overestimation of cost, we used published costs estimates from public facilities as opposed to private facilities. Future costing studies for the eight diseases not costed would enable assessment of the full health and economic benefit arising from implementation of the population-based interventions modelled. On balance, our study is likely to have underestimated healthcare cost savings.

We did not include industry costs in our evaluation due to lack of data on estimates of industry costs that may arise from the two regulatory interventions. This may mean that we underestimated the true cost of the regulatory interventions. However, in most instances, food labels may already exist with other details on them such as price, expiry date. The industry will only be adding information on kilojoules and possibly a health message on the labels using existing technology and this may not be expensive. For the tax on sugar-sweetened beverages, industry is continually reformulating products either voluntarily or due to mandatory measures and often already have funds set aside for reformulation. In addition, advancement in technology has allowed for less costly reformulation processes than seen in previous years. A key limitation was that the intervention scenario on increasing consumption of indigenous healthy foods was excluded from the cost effectiveness analysis. As reflected by the modelled interventions for this (a change in consumption levels related to supermarket food purchase and change in national consumption levels back to the 1975 average levels of energy intake), the indigenous foods intervention would have a gigantic effect. However, there is little or no evidence, and the outlined scenario in Fig. 1 is highly unlikely particularly for the change in national consumption levels. We also were not able to determine the full cost of the hypothetical scenario under the indigenous food intervention. However, we provide a record of the costing process with feasible estimates for as many aspects as was possible.

### Implications for policy and future research

Though there are several national health strategies in Kenya that guide the prevention of overweight and obesity and related NCDs [17–19], most of the government policy actions are still at the development stage [30]. Our study provides evidence on potential health and economic benefits and cost effectiveness of interventions that Kenya could consider for the prevention of overweight and obesity in the country.

The first intervention is an extensive proposal for research on Kenya’s indigenous foods. We did not identify any previous research that investigates the effect of increased consumption of healthy indigenous foods on either kilojoule intake, body weight or body mass index for a population. This is an area for future research. Nonetheless, our modelled two scenarios indicate that this intervention has potential for substantial health and economic benefits for the entire population.

Implementation of the mandatory kilojoule labelling on food served in formal sector restaurants and tax on sugar-sweetened beverages interventions in Kenya could be complemented by health education campaigns to the public on healthy diets particularly on daily recommendation energy and sugar levels. An aspect to consider would be the combination of the interventions. If the two regulatory interventions were implemented at the same time, the health education could cover awareness creation for both aspects of healthy diet. This also means that potentially more health gains and cost savings would be expected. To model the actual impact estimates of combined interventions, we would require evidence of (combined) effect. Future research that estimates the joint effects could help identify possible intervention packages that policy makers could consider.

Considering the COVID-19 pandemic, recent evidence shows that weight gain was reported during the pandemic [97]. This may lead to a greater proportion of the population having overweight or obesity. The effect of interventions on consumption and BMI would not differ. However, the health impact would be greater because a greater proportion of the BMI distribution is in the ‘danger zone’ in the exponential risk curve.

Though specific considerations and further research on the proposed interventions is required, most evidence will be gathered upon implementation of the interventions and data collection or through experimental or pilot studies in Kenya or elsewhere. Examples of such considerations include determination of whether people read and understand the labels on the foods served in formal sector restaurants, whether different labelling mechanisms are required for dine in customers and those who take away and inclusion of appropriate health messaging including pictorials.

Placing results from cost-effectiveness analyses within a broader framework that incorporates other factors (implementation considerations) that are important to decision-makers improves the relevance to policy and priority setting [20, 28, 77]. Future studies could incorporate the assessment of these factors in relation to our modelled interventions.

Healthy eating has been linked to sustainable environments [98]. Hence, interventions that create healthy food environments in Kenya are also likely to contribute to food security and protect against climate change. Also, additional benefits could be derived if the government used the revenue generated from the SSB tax to improve the health and wellbeing of the citizens. In turn, this could enhance acceptability of the tax intervention by the public and other key stakeholders. Future priority setting studies should include these factors as part of the other implementation considerations.

Subject to data availability, future studies could assess the impact and cost effectiveness of interventions at the county (sub country) level in Kenya and stratify analyses based on socioeconomic factors such as education level, wealth quintiles, urban versus rural residence.

In relation to cost-effectiveness, there is emerging discussion that ICERs should be compared to cost-effectiveness thresholds based on the best estimates of opportunity cost of health care spending and not the consumption value of health [99, 100]. Woods et al. and Ochalek et al. provided indicative estimates of cost-effectiveness thresholds for Kenya on the basis of opportunity costs [99, 100]. However, the estimates were based on limited data and strong uncertain assumptions. The authors rightly attribute this to the lack of attention paid to estimating opportunity cost of health care spending in the literature to date. This is an area of future research.

Stakeholders engaged in our study had proposed a total of 24 broad strategies for the prevention of overweight and obesity in Kenya [26]. Due to time limitation we included the two highest ranked broad strategies in this study. Also, we did not assess any physical activity related interventions in this study. Future research should evaluate additional preventive strategies particularly those that create supportive environments that make the choice of healthier foods and regular physical activity the easiest choice [101]. Such evaluations will provide additional evidence that informs priority setting for NCD control in Kenya and similar settings.

## Conclusion

All interventions evaluated in this study yielded health gains, healthcare cost savings and productivity gains. The lifetime productivity gains estimated were greater than the corresponding healthcare costs savings. In both main and sensitivity analysis, the two interventions assessed for cost effectiveness were found to be very cost effective (without cost offsets) and dominant (with cost offsets included), i.e., from a health sector perspective. These interventions should be given consideration for implementation as part of Kenya’s NCD control plans.

### Electronic Supplementary Material

Below is the link to the electronic supplementary material

Supplementary Material 1

Supplementary Material 2

## Data Availability

Data generated or analysed during this study are included in this published article [and its supplementary information file]. Additional datasets generated during and/or analysed during the current study are available from the corresponding author on reasonable request.

## List of Abbreviations

ACE: Assessing cost-effectiveness
BMI: Body mass index
CKD: Chronic kidney disease
DALYs: Disability adjusted life years
GDP: Gross domestic product
HALYS: Health-adjusted life years
ICERs: Incremental cost-effectiveness ratios
LMICs: Low- and middle-income countries
NCDs: Non-communicable diseases
pMSLT: Proportional multi-state lifetable
SF: Supplementary File
SSB: Sugar-sweetened beverages
T2DM: Type 2 diabetes
UI: Uncertainty interval
USD: United States Dollar
WHO: World Health Organization
