# Supplementary material for: The health and economic impact and cost effectiveness of interventions for the prevention and control of overweight and obesity in Kenya: a stakeholder engaged modelling study
Source: Cost Eff Resour Alloc. 2023 Sep 21;21:69. doi: 10.1186/s12962-023-00467-3 (PMC10512507; doi:10.1186/s12962-023-00467-3)
Supplement: Supplementary file 1 — Supplementary Material 1 [file 12962_2023_467_MOESM1_ESM.docx]

**Supplementary file**

**Article title:** The health and economic impact and cost effectiveness of interventions for the prevention and control of overweight and obesity in Kenya: a stakeholder engaged modelling study

**Corresponding author**

**Dr. Mary Njeri Wanjau**

Griffith University, School of Medicine & Dentistry, Gold Coast, Queensland, Australia.

Gold Coast campus, Parklands Drive, Southport, QLD, 4222

[m.wanjau@griffith.edu.au](mailto:m.wanjau@griffith.edu.au)

University of Nairobi, School of Nursing Sciences, Nairobi, Kenya.

ORCID**:** 0000-0002-4588-7528

**Co-authors**

**Dr. Lucy W. Kivuti-Bitok**

University of Nairobi, School of Nursing Sciences, Nairobi, Kenya.

P.O. Box 19676-00200, Nairobi, Kenya

[lukibitok@uonbi.ac.ke](about:blank)

ORCID**:** 0000-0002-0958-1580

**Dr. Leopold N. Aminde**

Griffith University, School of Medicine & Dentistry, Gold Coast, Queensland, Australia.

Non-communicable Disease Unit, Clinical Research Education, Networking & Consultancy, Douala, Cameroon.

[l.aminde@griffith.edu.au](about:blank)

ORCID**:** 0000-0003-2787-7518

**Prof. J. Lennert Veerman**

Griffith University, School of Medicine & Dentistry, Gold Coast, Queensland, Australia.

[l.veerman@griffith.edu.au](about:blank)

ORCID**:** 0000-0002-3206-8232

Table of Contents

[1. Methods 3](#_Toc142134517)

[1.1 BMI trend 3](#_Toc142134518)

[1.2 Average height measures 3](#_Toc142134519)

[1.3 Definition of interventions and evidence of effect from literature 4](#_Toc142134520)

[1.3.1 Age and sex specific effect size modelled expressed as a change in BMI units 4](#_Toc142134521)

[1.3.2 Broad strategy 1: Increased consumption of healthy indigenous foods 4](#_Toc142134522)

[1.3.3 Broad strategy 2: Interventions that Kenya could implement towards creation of healthy food environment 8](#_Toc142134523)

[1.4 Estimation of intervention costs 12](#_Toc142134524)

[1.5 Estimation of healthcare costs 14](#_Toc142134525)

[1.6 The 2019 Kenya population 17](#_Toc142134526)

[1.7 Productivity gains 17](#_Toc142134527)

[2. Additional results from main analysis 19](#_Toc142134528)

[2.1 A 20% tax on sugar sweetened beverages 20](#_Toc142134529)

[2.2 Mandatory kilojoule menu labelling 23](#_Toc142134530)

[2.3 Change in consumption levels related to supermarket food purchase 27](#_Toc142134531)

[2.4 Change in national consumption levels back to the 1975 average level of energy intake 30](#_Toc142134532)

[3. References 33](#_Toc142134533)

# Methods

## 1.1 BMI trend


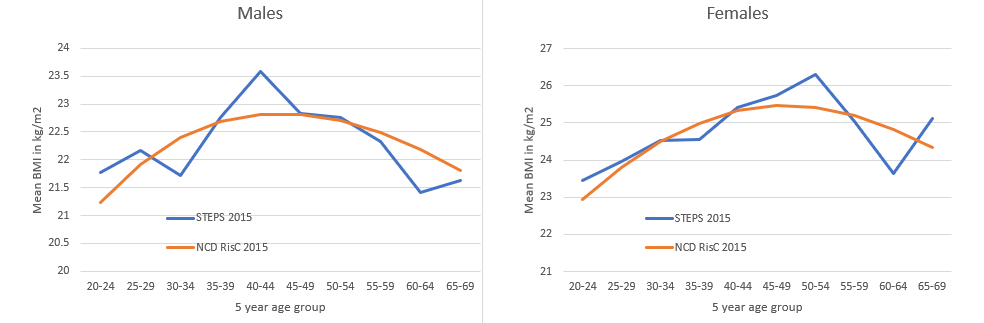


Figure 1: Mean adult BMI levels from the 2015 Kenya STEPS survey and the 2015 estimates from the NCD- RisC study

This figure is an illustration of our finding that the measured mean (and standard deviation) BMI levels in Kenya from 1993 to 2015 national survey data [1-6] are comparable with the corresponding BMI levels from the NCD- RisC study (mean and lower, upper confidence intervals).


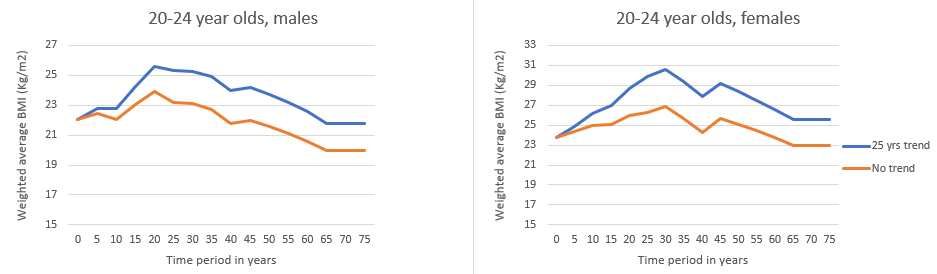


Figure 2 : Weighted average BMI for each time period for the 20–24-year-old age group

A linear trend is applied. This figure presents an example of the weighted average BMI predicted for the 20-24-year-old age group in future years.

## 1.2 Average height measures

Table 1: Average height by age and sex for Kenya population

| **Age group** | **Male** | **Female** |
| --- | --- | --- |
| 20-39 | 1.7011 | 1.5976 |
| 40-59 | 1.7063 | 1.5944 |
| 60-69 | 1.6794 | 1.5749 |

Data derived from the Kenya ‘STEPwise approach to Surveillance of NCD risk factors’ (STEPS) survey [7].

##

## 1.3 Definition of interventions and evidence of effect from literature

### 1.3.1 Age and sex specific effect size modelled expressed as a change in BMI units

For each modelled intervention, we present the age and sex specific effect size expressed as a change in BMI units.

Table 2 : Effect size expressed as a change in BMI units

| **5-year age group** | **20% SSB tax** | | **Mandatory kj menu Labelling** | | **Change in consumption levels related to supermarket food purchase** | | **Change in national consumption levels back to the 1975 average levels of energy intake** | |
| --- | --- | --- | --- | --- | --- | --- | --- | --- |
|  | Males | Females | Males | Females | Males | Females | Males | Females |
|  | (∆ BMI) | (∆ BMI) | (∆ BMI) | (∆ BMI) | (∆ BMI) | (∆ BMI) | (∆ BMI) | (∆ BMI) |
| 20-24 | -0.0512 | -0.0494 | -0.0299 | -0.0339 | -0.9663 | -0.9663 | -4.3223 | -4.9002 |
| 25-29 | -0.0525 | -0.0507 | -0.0299 | -0.0339 | -0.9663 | -0.9663 | -4.3223 | -4.9002 |
| 30-34 | -0.0517 | -0.0501 | -0.0299 | -0.0339 | -0.9663 | -0.9663 | -4.3223 | -4.9002 |
| 35-39 | -0.0498 | -0.0483 | -0.0299 | -0.0339 | -0.9663 | -0.9663 | -4.3223 | -4.9002 |
| 40-44 | -0.0468 | -0.0460 | -0.0297 | -0.0340 | -0.9663 | -0.9663 | -4.2959 | -4.9200 |
| 45-49 | -0.0438 | -0.0431 | -0.0297 | -0.0340 | -0.9663 | -0.9663 | -4.2959 | -4.9200 |
| 50-54 | -0.0406 | -0.0401 | -0.0297 | -0.0340 | -0.9663 | -0.9663 | -4.2959 | -4.9200 |
| 55-59 | -0.0377 | -0.0372 | -0.0297 | -0.0340 | -0.9663 | -0.9663 | -4.2959 | -4.9200 |
| 60-64 | -0.0363 | -0.0356 | -0.0307 | -0.0349 | -0.9663 | -0.9663 | -4.4346 | -5.0426 |
| 65-69 | -0.0339 | -0.0333 | -0.0307 | -0.0349 | -0.9663 | -0.9663 | -4.4346 | -5.0426 |
| 70-74 | -0.0318 | -0.0314 | -0.0307 | -0.0349 | -0.9663 | -0.9663 | -4.4346 | -5.0426 |
| 75-79 | -0.0301 | -0.0297 | -0.0307 | -0.0349 | -0.9663 | -0.9663 | -4.4346 | -5.0426 |
| 80-84 | -0.0286 | -0.0282 | -0.0307 | -0.0349 | -0.9663 | -0.9663 | -4.4346 | -5.0426 |
| 85-89 | -0.0273 | -0.0269 | -0.0307 | -0.0349 | -0.9663 | -0.9663 | -4.4346 | -5.0426 |
| 90+ | -0.0260 | -0.0258 | -0.0307 | -0.0349 | -0.9663 | -0.9663 | -4.4346 | -5.0426 |

Footnote: The resulting BMI change is sex and age specific due to the average height measures by age and sex for the Kenya population (SF Table 1). The effect sizes in the table reflect the modelled base case scenarios.

###

### 1.3.2 Broad strategy 1: Increased consumption of healthy indigenous foods

We searched PubMed and Scopus electronic databases for relevant literature. Our key search terms covered two concept areas: indigenous foods and body mass index (Table 2). We conducted the search on the 4^th^ of August 2022 and restricted it to research articles published in English within the last 10 years.

Table 3: Search algorithm and results

| **Database** | **Search algorithm** | **Results** |
| --- | --- | --- |
| PubMed | ("indigenous food*"[Title] OR "traditional food*"[Title] OR indigenous diet[Title] OR traditional diet[Title] OR diet[Title]) AND (effect[Title] OR "body mass"[Title] OR "body mass index"[Title] OR "body weight"[Title] OR weight[Title]) AND ("2012/08/07"[PDat] : "2022/08/04"[PDat]) | 2,167 |
| Scopus | ( TITLE-ABS-KEY ( "indigenous food*"  OR  "traditional food*"  OR  indigenous  AND diet  OR  traditional  AND diet  OR  diet )  AND  TITLE-ABS-KEY ( "body mass"  OR  "body mass index"  OR  "body weight"  OR  weight ) )  AND  ( LIMIT-TO ( PUBYEAR ,  2022 )  OR  LIMIT-TO ( PUBYEAR ,  2021 )  OR  LIMIT-TO ( PUBYEAR ,  2020 )  OR  LIMIT-TO ( PUBYEAR ,  2019 )  OR  LIMIT-TO ( PUBYEAR ,  2018 )  OR  LIMIT-TO ( PUBYEAR ,  2017 )  OR  LIMIT-TO ( PUBYEAR ,  2016 )  OR  LIMIT-TO ( PUBYEAR ,  2015 )  OR  LIMIT-TO ( PUBYEAR ,  2014 )  OR  LIMIT-TO ( PUBYEAR ,  2013 )  OR  LIMIT-TO ( PUBYEAR ,  2012 )  OR  LIMIT-TO ( PUBYEAR ,  2011 ) )  AND  ( LIMIT-TO ( DOCTYPE ,  "ar" )  OR  LIMIT-TO ( DOCTYPE ,  "re" ) )  AND  ( LIMIT-TO ( LANGUAGE ,  "English" ) ) | 577 |

A total of 2,744 articles were identified (2,167 from PubMed and 577 from Scopus). An additional eight articles were identified by use of the function ‘similar articles’ on PubMed database. After title/abstract screening and removal of duplicates, 37 articles were included for full text review. Upon review of the full text articles, we considered eight articles to be related to the review question [8-15]. Still, none of the eight studies provided empirical evidence on the effect of increased consumption of healthy indigenous foods on either energy intake, body weight or body mass index. We present a summary of the eight studies in Table 3.

Table 4: Summary of findings from articles included in full text review

| **#** | **Study** | **Summary** |
| --- | --- | --- |
| #1 | Greger 2020 [8] | A review article that presents current evidence on whole food, plant-based diet for weight loss. A diet centred on whole plant foods is recommended as a sustainable solution to the obesity epidemic. |
| #2 | Papadaki 2020 [9] | A Systematic Review and Meta-Analysis of Controlled Trials investigating the effect of the Mediterranean Diet on a multitude of outcomes related to metabolic health in adults. They assessed the effect on several metabolic syndrome risk factors that included body weight and body mass index. In 40 included studies (12,571 participants), they found an effect of the Mediterranean diet on body weight (-1.72 kg mean difference, 95% CI -2.40, -1.05). In 37 included studies (5,679 participants), they found an effect of the Mediterranean diet compared with control diets on BMI (-0.41 kg/m2 mean difference, 95% CI -0.71, -0.10). |
| #3 | Motswagole 2020 [10] | This was a cross-sectional survey in urban and rural areas nationwide in Botswana. Authors looked at the ‘Association of General and Central Obesity with Dietary Patterns and Socioeconomic Status in Adult Women in Botswana’. They found that individuals in the highest tertile of the Botswana traditional food pattern had a significantly higher risk of general obesity (RR = 1.40, 95% CI: 1.07–1.84) and central obesity (RR = 1.20, 95% CI: 0.97–1.48). Authors concluded that the Botswana traditional food pattern, characterised by a high carbohydrate intake, was found to be associated with a high risk of obesity in this study. The proportion of obesity was highest among women with low education. The authors lined this finding to the existence of food basket for various programs that target low socioeconomic class citizens in Botswana contain mainly sorghum meal, maize meal, vegetable oil, beans, and skimmed milk, which may be contributing to body weight gain in this population group. In comparison, our intervention is about researching and promoting the healthier varieties of traditional foods in Kenya. |
| #4 | Vogliano 2021 [11] | This was an observational mixed-method study that assessed nutrition transitions and diet quality by comparing three geographically unique rural and urban indigenous Solomon Islands populations. They found that participants in rural areas sourced more energy from wild and cultivated foods; consumed a wider diversity of foods and had significantly lower body fat, waist circumference, and body mass index (BMI) when compared to urban populations. |
| #5 | Zhu 2021 [12] | In this secondary analysis authors assess longitudinal associations of an overall plant-based diet and specific plant foods with weight-loss maintenance and cardiometabolic risk factors. They analysed longitudinal data on participants with overweight or obesity and pre-diabetes from a 3-year weight-loss maintenance phase of a randomized trial study. The findings showed that the plant-based diet index was inversely associated with weight regain. The authors conclude that an overall plant-based diet may improve weight management. |
| #6 | Agnoli 2018 [13] | The study investigated associations between adherence to a Mediterranean diet and long-term changes in weight and waist circumference in volunteers recruited to the Italian section of the prospective European Prospective Investigation into Cancer and Nutrition (EPIC). They found that adherence to a traditional Italian Mediterranean diet may help prevent weight gain and abdominal obesity. High adherence was associated with reduced risk of becoming overweight/obese (OR 0.91, 95% CI 0.84–0.99); and lower risk of abdominal obesity (OR 0.91, 95% CI 0.84–0.99). |
| #7 | Leonetti 2016 [14] | In this study, a sample of 284 patients were randomized into 2 groups: group A (n = 142) followed a basic traditional Chinese diet (BCTD), and group B (n = 142) followed a Western standard diet (WSD). The patients enrolled were compared before treatment and 6 weeks after treatment, and then follow-ups were made at 1 year and 5 years. In the BCTD group, BMI decreased by 0.46 kg/m^2^, versus respectively 0.28 kg/m^2^ in the WSD group. Outcomes showed that the BCTD had a better effect on BMI. |
| #8 | Liguori 2013 [15] | In this trial, a total of 694 enrolled subjects were divided into two groups: group A undergoing a 1200-kcal BTCD, and group B undergoing a 1200-kcal standard western diet. On 6 weeks after treatment, patients in group A lost more weight (0.37 +/- 0.52) kg than group B (0.26 +/- 0.79) kg. The results showed that the two diets could lead to lower BMI. However, the BTCD was significantly better than the western standard diet. |

To model the possible effect that could arise from increased consumption of healthy indigenous foods, we built two intervention scenarios underpinned by evidence that changes in the global food system are the main drivers of the rise of the global obesity epidemic [16]. Specifically, in Sub-Saharan African countries, including Kenya, the changes in diet are characterised by a departure from traditional foods and have been associated with high BMI [17-20].

*First possible intervention scenario: Change in consumption levels related to supermarket food purchase*

For our second possible effect scenario, we conducted broader searches to scope for any additional relevant literature that may be available. We identified a study that investigated whether supermarkets indirectly contributed to overweight [21] and another that investigated the effects of purchasing food in supermarkets on people’s BMI [22]. Both are Kenyan studies that utilised cross-sectional data. Kimenju and colleagues found that buying food in a supermarket was associated with a higher probability of overweight in adults [21]. Findings by Demmler et al. indicated that purchasing food in supermarkets contributed to a higher BMI (+ 1.82 [SE 0.24] kg/m^2^) [22].

*Second intervention scenario: Change in national consumption levels* back to the 1975 average levels of energy intake

In our first possible effect scenario we used data from the Food and Agriculture Organization (FAO) for Sub-Sahara Africa, which shows an increase in total food consumption (in kcal/person/day) from 1964 projected to 2030 (Table 4) [23]. We modelled a scenario where the Kenya population reverted to the 1975 levels of food consumption of 2,079 kcal/person/day and compared it to the 2015 levels (taken to represent estimate 2019 levels). The 1975 consumption would be expected to reflect a higher consumption of indigenous foods. However, one key consideration is required for this scenario. Over the years, the prevalence of underweight in Kenya adults has decreased (Table 5) [7, 24]. A return to the 1975 consumption levels in our scenario is on the assumption that only overweight and obese individuals would reduce intake hence not a return to high undernutrition rates. This assumption is supported by the recommended energy intake requirements for healthy adults (2,300 kcal/day for men and 1,900 kcal/day for women) [25].

Table 5: Changes in food consumption for the Sub-Saharan Africa region

| **Year** | **Total food consumption (kcal/person/day)** |
| --- | --- |
| 1964/66 | 2,057 |
| 1974/76 | 2,079 |
| 1984/86 | 2,058 |
| 1997/99 | 2,195 |
| 2015 | 2,360 |
| 2030 | 2,540 |

Source: Food and Agriculture Organization (FAO), 2003 [23].

Kcal: kilocalories

Sub-Saharan data on total food consumption reported by Food and Agriculture Organization (FAO) shows an increase in consumption (in kcal/ person/day from 1964 projected to 2030.

Table 6: Prevalence of underweight in adults in Kenya

| **Age-standardised global prevalence of underweight [24]** | | | | | |
| --- | --- | --- | --- | --- | --- |
| **BMI <18.5 kg/m^2^** | | | | | |
| **Sex** | **Year** | | | | |
|  | **1975** | **1985** | **1995** | **2005** | **2014** |
| Men ^a, b^ | 0.195  (0.081-0.325) | 0.179  (0.091-0.283) | 0.163  (0.084-0.258) | 0.151  (0.077-0.243) | 0.133  (0.056-0.237) |
| Women ^a, b^ | 0.171  (0.075-0.289) | 0.152  (0.082-0.236) | 0.133  (0.077-0.204) | 0.116  (0.066-0.180) | 0.099  (0.047-0.172) |
| ^a^ Percentage prevalence reported  ^b^ Numbers in brackets show 95% credible intervals | | | | | |
| **Percentage underweight (<18.5 kg/m^2^) from the latest national survey, 2015 [7]** | | | | | |
| **Sex** | **Mean (%)** | **95% confidence intervals** | | | |
| Men | 13.7 | 10.3-17.0 | | | |
| Women | 8.9 | 6.5-11.4 | | | |
| Both | 11.3 | 9.0-13.7 | | | |

Source of data indicated in the cited references [7, 24]

### 1.3.3 Broad strategy 2: Interventions that Kenya could implement towards creation of healthy food environment

*Intervention: Tax on sugar-sweetened beverages (SSBs)*

An SSB was defined as a non-alcoholic drink with added sugar, including carbonated soft drinks, concentrates, energy drinks and flavoured mineral waters [26]. The tax was assumed to apply in addition to the existing Goods and Services Tax (GST), with the effect that the consumer price of SSBs would increase by 20%. In our analysis, we assumed that producers pass on the price increase in full to the consumers. A review of empirical evidence suggests that SSB taxes are passed on in full when they are applied to entire countries (but not necessarily when applied to smaller areas) [27, 28]. Price elasticity indicates how price influences demand. An ‘own-price’ elasticity of -1 indicates that for every percent increase in price, demand drops by 1%. ‘Cross-price elasticity’ is the change in demand for a product [in our case, milk, diet drinks and fruit juice] because of a price change of another product [in our study, SSBs]). We did not find evidence on cross price elasticity from Kenya or similar setting. However using available evidence (SF Table 7) [29], in our sensitivity analysis, we assessed the impact that ‘cross-price elasticities’ would have on the outcomes.

Table 7 : Price elasticity and ‘cross-price elasticities

| **Price elasticities ^a^** | **Own-price elasticity** | **Cross price elasticity** | | |
| --- | --- | --- | --- | --- |
|  | **SSB** | **Fruit juice** | **Milk** | **Diet drinks** |
| **Mean** | -1.198 | 0.215 | 0.087 | -0.303 |
| **LCI** | -1.340 | 0.140 | 0.034 | -0.626 |
| **HCI** | -1.057 | 0.289 | 0.139 | 0.019 |

^a^ Source of data: based on 2015 updated price elasticity values of a global systematic review and meta-analysis of studies in the USA, Mexico, Brazil, and France [29]. (Uncertainty distribution in the model: normal)

The estimates of current (baseline) dietary intake data for Kenya (SF Table 8) were sourced from Euromonitor International and Global Dietary Database [26, 30]. Euromonitor International reported the total volume of sugar-sweetened beverages (SSB) consumption in Kenya from the year 2007 projected to 2026. We fitted a linear trend to the Euromonitor data to determine the absolute consumption change in litres/person/day. In the model, we applied this trend to SSB consumption for 15 years into the future using the year 2019 as our baseline. For modelling purposes, we used the latest available age and sex specific consumption levels for SSB, juice and milk in Kenya provided by Global Dietary Database (GDD) in grams/day/person for the year 2018 (taken to represent 2019 consumption levels in our model). We applied a correction to downscale the GDD data (to 97%) arriving at the total sales of SSBs reported in the Euromonitor on the assumption that this would be the quantity in the formal market affected by tax. We used the 2019 total sales price and volumes reported in Euromonitor to derive the retail selling price per litre for SSB, fruit juice and milk.

Table 8: Estimates of consumption data for Kenya in litres/day/person

| **Sex/5-year age group** | **SSB** | | | **Juice** |  |  | **Milk** |  |  |
| --- | --- | --- | --- | --- | --- | --- | --- | --- | --- |
|  | **Mean** | **LCI** | **HCI** | **Mean** | **LCI** | **HCI** | **Mean** | **LCI** | **HCI** |
| **Men** |  |  |  |  |  |  |  |  |  |
| 20-24 | 0.0382 | 0.0223 | 0.0650 | 0.0197 | 0.0147 | 0.0265 | 0.0861 | 0.0748 | 0.0984 |
| 25-29 | 0.0393 | 0.0229 | 0.0673 | 0.0188 | 0.0133 | 0.0264 | 0.0754 | 0.0653 | 0.0868 |
| 30-34 | 0.0387 | 0.0224 | 0.0665 | 0.0174 | 0.0118 | 0.0255 | 0.0694 | 0.0598 | 0.0804 |
| 35-39 | 0.0370 | 0.0214 | 0.0637 | 0.0159 | 0.0104 | 0.0240 | 0.0676 | 0.0579 | 0.0787 |
| 40-44 | 0.0346 | 0.0200 | 0.0600 | 0.0144 | 0.0093 | 0.0223 | 0.0696 | 0.0596 | 0.0816 |
| 45-49 | 0.0321 | 0.0184 | 0.0552 | 0.0131 | 0.0084 | 0.0206 | 0.0752 | 0.0639 | 0.0890 |
| 50-54 | 0.0293 | 0.0168 | 0.0507 | 0.0120 | 0.0075 | 0.0188 | 0.0846 | 0.0708 | 0.1020 |
| 55-59 | 0.0268 | 0.0151 | 0.0468 | 0.0109 | 0.0067 | 0.0175 | 0.0978 | 0.0796 | 0.1209 |
| 60-64 | 0.0245 | 0.0134 | 0.0439 | 0.0100 | 0.0059 | 0.0168 | 0.1138 | 0.0893 | 0.1452 |
| 65-69 | 0.0226 | 0.0119 | 0.0414 | 0.0092 | 0.0052 | 0.0161 | 0.1314 | 0.0996 | 0.1739 |
| 70-74 | 0.0208 | 0.0107 | 0.0391 | 0.0085 | 0.0046 | 0.0157 | 0.1503 | 0.1095 | 0.2072 |
| 75-79 | 0.0194 | 0.0097 | 0.0372 | 0.0079 | 0.0040 | 0.0153 | 0.1704 | 0.1191 | 0.2429 |
| 80-84 | 0.0181 | 0.0087 | 0.0359 | 0.0074 | 0.0036 | 0.0151 | 0.1919 | 0.1297 | 0.2833 |
| 85-89 | 0.0170 | 0.0078 | 0.0349 | 0.0070 | 0.0032 | 0.0149 | 0.2145 | 0.1399 | 0.3260 |
| 90+ | 0.0159 | 0.0070 | 0.0339 | 0.0065 | 0.0029 | 0.0148 | 0.2372 | 0.1503 | 0.3686 |
| **Women** |  |  |  |  |  |  |  |  |  |
| 20-24 | 0.0316 | 0.0193 | 0.0516 | 0.0217 | 0.0160 | 0.0288 | 0.0940 | 0.0828 | 0.1069 |
| 25-29 | 0.0326 | 0.0201 | 0.0531 | 0.0206 | 0.0144 | 0.0288 | 0.0824 | 0.0721 | 0.0946 |
| 30-34 | 0.0322 | 0.0196 | 0.0523 | 0.0190 | 0.0127 | 0.0280 | 0.0757 | 0.0660 | 0.0875 |
| 35-39 | 0.0308 | 0.0186 | 0.0502 | 0.0174 | 0.0113 | 0.0263 | 0.0738 | 0.0641 | 0.0855 |
| 40-44 | 0.0289 | 0.0173 | 0.0472 | 0.0158 | 0.0101 | 0.0243 | 0.0759 | 0.0657 | 0.0882 |
| 45-49 | 0.0267 | 0.0161 | 0.0439 | 0.0144 | 0.0091 | 0.0224 | 0.0821 | 0.0704 | 0.0964 |
| 50-54 | 0.0244 | 0.0147 | 0.0401 | 0.0131 | 0.0082 | 0.0207 | 0.0925 | 0.0779 | 0.1109 |
| 55-59 | 0.0223 | 0.0132 | 0.0373 | 0.0119 | 0.0073 | 0.0193 | 0.1070 | 0.0869 | 0.1321 |
| 60-64 | 0.0204 | 0.0119 | 0.0352 | 0.0109 | 0.0065 | 0.0182 | 0.1242 | 0.0974 | 0.1592 |
| 65-69 | 0.0187 | 0.0105 | 0.0329 | 0.0101 | 0.0058 | 0.0175 | 0.1433 | 0.1075 | 0.1918 |
| 70-74 | 0.0173 | 0.0094 | 0.0312 | 0.0093 | 0.0051 | 0.0171 | 0.1639 | 0.1185 | 0.2276 |
| 75-79 | 0.0160 | 0.0085 | 0.0299 | 0.0087 | 0.0045 | 0.0167 | 0.1855 | 0.1293 | 0.2671 |
| 80-84 | 0.0149 | 0.0076 | 0.0290 | 0.0081 | 0.0039 | 0.0163 | 0.2086 | 0.1400 | 0.3109 |
| 85-89 | 0.0140 | 0.0068 | 0.0283 | 0.0076 | 0.0035 | 0.0162 | 0.2324 | 0.1512 | 0.3563 |
| 90+ | 0.0132 | 0.0062 | 0.0273 | 0.0072 | 0.0031 | 0.0161 | 0.2570 | 0.1624 | 0.3966 |

LCI: 95% lower confidence intervals, SSB: sugar-sweetened beverages, UCI: 95% upper confidence intervals

Baseline consumption for diet drinks was considered zero as no consumption levels were reported.

In SF Table 9 we present the consumption levels with trend applied for 15 years and levels after the intervention in kJ/day/person.

Table 9: Baseline consumption levels with trend applied for 15 years and levels after the intervention in kJ/day/person

| **Sex/5-year age group** | **Energy consumption (kJ/day/person) with trend applied for 15 years** | | | | | **Consumption after intervention (kJ/day/person)** | | | | |
| --- | --- | --- | --- | --- | --- | --- | --- | --- | --- | --- |
|  | **SSB** | **Fruit juice** | **Milk** | **Diet drinks** | **Total** | **SSB** | **Fruit juice** | **Milk** | **Diet drinks** | **Total** |
| **Men** |  |  |  |  |  |  |  |  |  |  |
| 20-24 | 70.95 | 35.91 | 229.08 | 0.00 | 335.94 | 57.02 | 35.91 | 229.08 | 0.00 | 322.01 |
| 25-29 | 72.73 | 34.16 | 200.61 | 0.00 | 307.50 | 58.46 | 34.16 | 200.61 | 0.00 | 293.23 |
| 30-34 | 71.70 | 31.59 | 184.61 | 0.00 | 287.90 | 57.63 | 31.59 | 184.61 | 0.00 | 273.83 |
| 35-39 | 69.03 | 28.91 | 179.92 | 0.00 | 277.87 | 55.48 | 28.91 | 179.92 | 0.00 | 264.32 |
| 40-44 | 65.23 | 26.30 | 185.01 | 0.00 | 276.54 | 52.43 | 26.30 | 185.01 | 0.00 | 263.74 |
| 45-49 | 61.11 | 23.89 | 199.90 | 0.00 | 284.90 | 49.11 | 23.89 | 199.90 | 0.00 | 272.90 |
| 50-54 | 56.59 | 21.76 | 225.01 | 0.00 | 303.36 | 45.49 | 21.76 | 225.01 | 0.00 | 292.25 |
| 55-59 | 52.63 | 19.87 | 260.03 | 0.00 | 332.54 | 42.30 | 19.87 | 260.03 | 0.00 | 322.20 |
| 60-64 | 48.97 | 18.20 | 302.65 | 0.00 | 369.82 | 39.35 | 18.20 | 302.65 | 0.00 | 360.21 |
| 65-69 | 45.79 | 16.72 | 349.55 | 0.00 | 412.07 | 36.80 | 16.72 | 349.55 | 0.00 | 403.08 |
| 70-74 | 42.97 | 15.48 | 399.91 | 0.00 | 458.36 | 34.54 | 15.48 | 399.91 | 0.00 | 449.93 |
| 75-79 | 40.64 | 14.44 | 453.32 | 0.00 | 508.41 | 32.67 | 14.44 | 453.32 | 0.00 | 500.43 |
| 80-84 | 38.59 | 13.52 | 510.43 | 0.00 | 562.54 | 31.02 | 13.52 | 510.43 | 0.00 | 554.96 |
| 85-89 | 36.81 | 12.67 | 570.54 | 0.00 | 620.02 | 29.58 | 12.67 | 570.54 | 0.00 | 612.79 |
| 90+ | 35.15 | 11.92 | 631.02 | 0.00 | 678.09 | 28.25 | 11.92 | 631.02 | 0.00 | 671.20 |
| **Women** |  |  |  |  |  |  |  |  |  |  |
| 20-24 | 60.37 | 39.48 | 250.11 | 0.00 | 349.96 | 48.52 | 39.48 | 250.11 | 0.00 | 338.11 |
| 25-29 | 61.93 | 37.47 | 219.07 | 0.00 | 318.47 | 49.77 | 37.47 | 219.07 | 0.00 | 306.31 |
| 30-34 | 61.30 | 34.60 | 201.43 | 0.00 | 297.33 | 49.27 | 34.60 | 201.43 | 0.00 | 285.30 |
| 35-39 | 58.99 | 31.59 | 196.18 | 0.00 | 286.76 | 47.41 | 31.59 | 196.18 | 0.00 | 275.18 |
| 40-44 | 55.99 | 28.81 | 201.91 | 0.00 | 286.71 | 45.00 | 28.81 | 201.91 | 0.00 | 275.72 |
| 45-49 | 52.46 | 26.20 | 218.30 | 0.00 | 296.96 | 42.16 | 26.20 | 218.30 | 0.00 | 286.66 |
| 50-54 | 48.78 | 23.79 | 245.93 | 0.00 | 318.50 | 39.20 | 23.79 | 245.93 | 0.00 | 308.93 |
| 55-59 | 45.30 | 21.70 | 284.68 | 0.00 | 351.68 | 36.41 | 21.70 | 284.68 | 0.00 | 342.79 |
| 60-64 | 42.30 | 19.89 | 330.46 | 0.00 | 392.65 | 34.00 | 19.89 | 330.46 | 0.00 | 384.34 |
| 65-69 | 39.57 | 18.32 | 381.27 | 0.00 | 439.16 | 31.80 | 18.32 | 381.27 | 0.00 | 431.39 |
| 70-74 | 37.33 | 16.96 | 435.92 | 0.00 | 490.21 | 30.00 | 16.96 | 435.92 | 0.00 | 482.88 |
| 75-79 | 35.30 | 15.79 | 493.45 | 0.00 | 544.53 | 28.37 | 15.79 | 493.45 | 0.00 | 537.61 |
| 80-84 | 33.49 | 14.77 | 554.84 | 0.00 | 603.10 | 26.92 | 14.77 | 554.84 | 0.00 | 596.53 |
| 85-89 | 31.95 | 13.84 | 618.21 | 0.00 | 664.01 | 25.68 | 13.84 | 618.21 | 0.00 | 657.73 |
| 90+ | 30.66 | 13.06 | 683.70 | 0.00 | 727.41 | 24.64 | 13.06 | 683.70 | 0.00 | 721.39 |

The change in consumption reported here is for the base case SSB tax scenario, i.e., 20% tax, 100% pass on rate and no cross-price elasticity applied.

*Intervention: Mandatory kilojoule labelling on food served in formal sector restaurants*

Currently, mandatory kilojoule labelling is in place in the US and several Australian states [31, 32]. Retail food establishments are required to display the number of calories for each standard food item and a clear statement concerning daily caloric intake on printed or online menus. Establishments covered by the legislation include chain restaurants (food businesses), large supermarkets, cafeterias, drinking places and similar retail food establishments that meet defined requirements.

We conducted a search on PubMed and Scopus databases to identify the latest comprehensive evidence on the effect of kilojoule labelling (on food served in formal sector restaurants) either on kilojoule intake, body weight or body mass index. We used key search terms that covered two concept areas: food labelling (calorie disclosure) and body mass index. We identified a meta-analysis where authors explored the effect of mandatory calorie exposure on both the retailers (41 studies) and consumers (186 studies) [33]. The majority of primary studies included were from the US and the rest from Canada, Australia and UK. The authors found that consumers (demand) selected 27.21 fewer calories per meal on average following disclosure and food retailers (supply) offered 15.34 lower calorie offerings per meal. They report that it was uncertain if the two effects entirely complemented each other and further context specific research on that would be required. In this study, we model an effect of kilojoule labelling on consumer consumption (27.21 fewer calories per meal) assuming that the effect on retailers’ supply is already considered in the estimate for change in consumer’s consumption.

## 1.4 Estimation of intervention costs

Table 10 : Costs estimates for various activities envisioned under the Research-based strategy that leads to increased production and consumption of healthy indigenous foods

| **Costing aspects** | **Costing components from Kenya food security budget & WHO NCD costing tool estimates** | **Cost in KShs.** | **Cost in USD** | **Costing time/ period from source** | **Cost in KShs. for each year** | **Cost in USD/year** | **Source** |
| --- | --- | --- | --- | --- | --- | --- | --- |
| **Broad strategy 1: Research-based strategy that leads to increased production and consumption of healthy indigenous foods from year 4, which leads to changes in body weight after the 5^th^ year.** | | | | | | | |
| Research fund | *Not costed* |  |  |  |  |  |  |
| Dissemination of research findings* | Components: Human resources, training, meetings, mass media, supplies & equipment, other | 747,380,210 | 7,327,882 | 2011 |  |  | WHO NCD costing tool [34] |
|  |  |  |  | Planning | 11,621,522 | 113,946 |  |
|  |  |  |  | Development | 59,324,874 | 581,666 |  |
|  |  |  |  | Partial implementation | 51,781,878 | 507,709 |  |
|  |  |  |  | 1^st^ year & 6^th^ year of full implementation | 53,416,576 | 523,737 |  |
|  |  |  |  | Other years of full implementation | 51,781,878 | 507,709 |  |
| Cost of promoting and increasing production of the healthy traditional diets | "Promote production of traditional high value and nutrient rich foods" | 250,000,000 | 2,451,190 | 2017-2022 | 50,000,000 | 490,238 | Kenya National food and nutrition security policy implementation framework 2017-2022 [35] |
|  | "Promote and support sustainable irrigation and water management systems" | 100,000,000 | 980,476 | 2017-2022 | 20,000,000 | 196,095 |  |
|  | "Improve accessibility to affordable farm inputs and credits" | 700,000,000 | 6,863,331 | 2017-2022 | 140,000,000 | 1,372,666 |  |
|  | "Support investment in infrastructure to increase food production and availability" | 400,000,000 | 3,921,903 | 2017-2022 | 80,000,000 | 784,381 |  |
| Increasing consumption of these foods through marketing, subsidies, or selected approaches | "Promote urban and peri urban agriculture to improve food availability and access for better nutrition" (This cost component considered as part of increasing access for consumption). | 100,000,000 | 980,476 | 2017-2022 | 20,000,000 | 196,095 |  |
| Increasing consumption of healthy indigenous foods through marketing* | Components: Human resources, training, meetings, mass media, supplies & equipment, other | 747,380,210 | 7,327,882 | 2011 |  |  | WHO NCD costing tool [34] |
|  |  |  |  | Planning | 11,621,522 | 113,946 |  |
|  |  |  |  | Development | 59,324,874 | 581,666 |  |
|  |  |  |  | Partial implementation | 51,781,878 | 507,709 |  |
|  |  |  |  | 1^st^ year & 6^th^ year of full implementation | 53,416,576 | 523,737 |  |
|  |  |  |  | Other years of full implementation | 51,781,878 | 507,709 |  |

*We considered the resources required for the implementation of the intervention ‘promote public awareness diet and physical activity’ costed in the NCD tool to be broadly similar to the intervention activity ‘Increasing consumption of healthy indigenous foods through marketing’ and ‘creation of awareness on research findings/ dissemination of research findings.

## 1.5 Estimation of healthcare costs

Table 11: 2019 Health care costs in USD

| **Age group** | **2019 Total health care costs in USD** | | | | **Costs of all other diseases, year 2019** | |
| --- | --- | --- | --- | --- | --- | --- |
|  | **Costs in USD** | | **Costs per person in USD** | | **Costs per person in USD** | |
|  | **Male** | **Female** | **Male** | **Female** | **Male** | **Female** |
| 0-4 | 211,311,453 | 297,171,638 | 65 | 94 | 61 | 89 |
| 5-14 | 100,841,169 | 141,815,008 | 16 | 23 | 10 | 16 |
| 15-24 | 120,840,100 | 169,939,915 | 22 | 31 | 15 | 22 |
| 25-34 | 130,257,586 | 183,183,919 | 34 | 46 | 22 | 31 |
| 35-44 | 171,101,963 | 240,624,205 | 63 | 86 | 39 | 61 |
| 45-54 | 262,208,203 | 368,748,782 | 153 | 218 | 101 | 169 |
| 55-64 | 404,422,822 | 568,748,122 | 401 | 557 | 290 | 465 |
| 65+ | 578,064,332 | 812,943,744 | 846 | 985 | 622 | 802 |
| **Total** | **1,979,047,628** | **2,783,175,333** |  |  |  |  |

To convert USD to Kenya shillings, use the world Bank’s official 2019 exchange rate of 102 [36].

Table 12: Per capita annual disease costs in USD

| **Disease modelled** | **Incident or prevalent case per year** | **Cost type/notes** | **Percentage of patients** | **Point estimates per year** | **Year of costing** | **Source** | **Country** |
| --- | --- | --- | --- | --- | --- | --- | --- |
| Asthma | Incident cost | Diagnosis |  | **4.23** | 2017 | Subramanian, S., et al., 2018 [37] | Kenya |
|  | Prevalent cost | Mild Asthma (management[mgt]/treatment[rx]) | 0.95 | 67.93 |  |  |  |
|  |  | Severe Asthma (mgt/rx) | 0.05 | 146.74 |  |  |  |
|  |  | Weighted average used as prevalent cost |  | **71.87** |  |  |  |
| Breast cancer | Incident cost | Breast cancer (clinical breast exam)(screening) |  | 3.90 | 2017 | Subramanian, S., et al., 2018 [37] | Kenya |
|  |  | Diagnosis |  | 401.00 |  |  |  |
|  |  | Total cost used as incident cost |  | **404.90** |  |  |  |
|  | Prevalent cost | Breast Cancer Treatment |  |  |  |  |  |
|  |  | Stage I | 0.07 | 1,340.38 | 2017 | Subramanian, S., et al., 2018 [37] | Kenya |
|  |  | Stage II | 0.35 | 1,340.38 |  |  |  |
|  |  | Stage III (curative approach) | 0.19 | 1,542.58 |  |  |  |
|  |  | Stage III (palliative approach) and Stage IV | 0.40 | 675.35 |  |  |  |
|  |  | Weighted average used as prevalent cost |  | **1,126.19** |  |  |  |
| Chronic Kidney diseases^a^ | Prevalent cost | Chronic kidney disease (dialysis) | 0.70 | 5,338.00 | 2017 | Subramanian, S., et al., 2018 [37] | Kenya |
|  |  | Chronic kidney disease (transplant) | 0.30 | 9,237.00 |  |  |  |
|  |  | Weighted average used as prevalent cost |  | **6,507.70** |  |  |  |
| Chronic lymphoid leukaemia (CLL) | Prevalent cost | Average cost of treatment |  | **1,164.20** | 2016 | Atieno et al.,2018 [38] | Kenya |
| Chronic myeloid leukaemia (CML) |  | Average cost of treatment |  | **686.00** |  |  |  |
| Acute lymphoid leukaemia (ALL) | Prevalent cost | We used total number of prevalent CLL and CML cases in Kenya from GBD to calculate a weighted average cost which was applied as the prevalent cost for ALL, AML and other Leukemia |  | **1,027.85** |  |  |  |
| Acute myeloid leukaemia (AML) |  |  |  |  |  |  |  |
| Other leukaemia |  |  |  |  |  |  |  |
| Multiple myeloma | Prevalent cost | Average cost of treatment |  | **1,473.10** | 2016 | Atieno et al.,2018 [38] | Kenya |
| Colon and rectum cancer | Prevalent cost | Average cost of treatment |  | **1,742.10** |  |  |  |
| Diabetes mellitus type 2 | Incident cost | Diabetes—random blood sugar (screening) |  | 4.95 | 2017 | Subramanian, S., et al., 2018 [37] | Kenya |
|  |  | Diagnosis |  | 41.95 |  |  |  |
|  |  | Total cost used as incident cost |  | **46.90** |  |  |  |
|  | Prevalent cost | Insulin only (mgt/rx) | 0.32 | 186.40 |  |  |  |
|  |  | Oral medication only (mgt/rx) | 0.25 | 88.61 |  |  |  |
|  |  | Both insulin and oral medication (mgt/rx) | 0.43 | 234.44 |  |  |  |
|  |  | Weighted average used as prevalent cost |  | **182.61** |  |  |  |
| Gallbladder and biliary tract cancer | Prevalent cost | Average cost of treatment |  | **407.90** | 2016 | Atieno et al.,2018 [38] | Kenya |
| Hypertensive heart disease | Prevalent cost | Annual treatment cost for hypertension |  |  |  |  |  |
|  |  | Treatment—1 drug | 0.20 | 25.64 | 2017 | Subramanian, S., et al., 2018 [37] | Kenya |
|  |  | Treatment—2 drug | 0.35 | 67.25 |  |  |  |
|  |  | Treatment—3 drug | 0.25 | 81.20 |  |  |  |
|  |  | Treatment—4 drug | 0.10 | 110.33 |  |  |  |
|  |  | Treatment—resistant | 0.10 | 159.36 |  |  |  |
|  |  | Weighted average used as prevalent cost |  | **75.93** |  |  |  |
| Intracerebral haemorrhage | Prevalent cost | Used the cost of prevalent haemorrhagic stroke |  | **1,406.30** | 2013-2017 | Aminde et al. 2021 [39] | Cameroon |
| Subarachnoid haemorrhage | Prevalent cost | Used the cost of prevalent haemorrhagic stroke |  | **1,406.30** | 2013-2017 | Aminde et al. 2021 [39] | Cameroon |
| Ischaemic heart disease^b^ | Incident cost | Acute myocardial infarction | 0.2F, 0.35M | 1,995.65 | 2017 | Subramanian, S., et al., 2018 [37] | Kenya |
|  |  | Angina | 0.35 | 1,236.81 |  |  |  |
|  |  | Heart failure (secondary to hypertension) | 0.10 | 1,026.07 |  |  |  |
|  |  | Weighted average calculated using the given event distribution as cited by authors. |  | **1,633.10** |  |  |  |
|  | Prevalent cost | All CHD states (chronic annual cost for secondary prevention) |  | **300.00** | 2012 | Gazianio et al., 2014 [40] | South Africa |
| Ischaemic stroke | Incident cost | Stroke cost per inpatient or outpatient episode |  | **1,873.93** | 2017 | Subramanian, S., et al., 2018 [37] | Kenya |
|  | Prevalent cost | Stroke (chronic annual cost for secondary prevention) |  | **900.00** | 2012 | Gazianio et al., 2014 [40] | South Africa |
| Kidney cancer | Prevalent cost | Average cost of treatment for renal carcinoma |  | **671.40** | 2016 | Atieno et al.,2018 [38] | Kenya |
| Liver cancer | Prevalent cost | Average cost of treatment for Liver cancer |  | **1,303.40** |  |  |  |
| Oesophageal cancer | Prevalent cost | Average cost of treatment |  | **866.80** |  |  |  |
| Ovarian cancer | Prevalent cost | Average cost of treatment |  | **2,217.00** |  |  |  |
| Pancreatic cancer | Prevalent cost | Average cost of treatment |  | **1,244.00** |  |  |  |
| Thyroid cancer | Prevalent cost | Average cost of treatment for follicular thyroid carcinoma |  | **1,342.70** |  |  |  |
| Uterine cancer | Prevalent cost | Average cost of treatment for endometrial carcinoma |  | **1,624.40** |  |  |  |
| ^a^Only estimated chronic kidney disease (CKD) cases on dialysis and transplant were costed in our model. Of the 9.1% global CKD prevalence, dialysis accounted for 0·041% (0·037 to 0·044), and kidney transplantation for 0·011% (0·010 to 0·012) [41]. ^b^We considered the acute presentations IHD as acute myocardial infarction, angina and heart failure (secondary to hypertension) [37]. To determine the number of people diagnosed with IHD that had experienced each of the above events (i.e., myocardial infarction, angina and heart failure [secondary to hypertension]), we used proportions identified in a study by Subramanian and colleagues [42] based on previous research [43, 44]. | | | | | | | |

## 1.6 The 2019 Kenya population

Table 13: Kenya population numbers for 2019

| **Age in years** | **Males** | **Females** | **Total** |
| --- | --- | --- | --- |
| Under 5 | 3,263,525 | 3,152,518 | 6,416,043 |
| 05-09 | 3,261,604 | 3,161,257 | 6,422,860 |
| 10-14 | 3,215,815 | 3,105,368 | 6,321,183 |
| 15-19 | 2,960,849 | 2,902,357 | 5,863,206 |
| 20-24 | 2,453,646 | 2,507,200 | 4,960,846 |
| 25-29 | 2,019,890 | 2,140,126 | 4,160,016 |
| 30-34 | 1,758,523 | 1,869,511 | 3,628,034 |
| 35-39 | 1,499,836 | 1,556,487 | 3,056,323 |
| 40-44 | 1,222,912 | 1,230,053 | 2,452,965 |
| 45-49 | 969,217 | 957,160 | 1,926,378 |
| 50-54 | 748,069 | 734,187 | 1,482,256 |
| 55-59 | 577,482 | 577,447 | 1,154,929 |
| 60-64 | 430,217 | 443,763 | 873,980 |
| 65-69 | 303,584 | 320,021 | 623,605 |
| 70-74 | 195,885 | 224,304 | 420,188 |
| 75-79 | 109,365 | 144,803 | 254,168 |
| 80-84 | 49,707 | 82,254 | 131,961 |
| 85-89 | 18,697 | 37,725 | 56,423 |
| 90-94 | 4,902 | 12,933 | 17,834 |
| 95+ | 1,003 | 3,508 | 4,511 |
| **TOTALS** | **25,064,727** | **25,162,982** | **50,227,709** |

We used age- and sex-specific estimates for the 2019 Kenya population from the Global Burden of Disease (GBD) 2019 study [45].

## 1.7 Productivity gains

We estimated productivity outcomes for the 2019 working population in Kenya (20 years up to retirement age of 65 years) over their lifetime. We used the Human Capital Approach [46], a methodology previously used in Nomaguchi et al. [47]. Productivity gains are estimated from the age of premature death until the age of traditional retirement in Kenya (65yrs). People obtain additional life years (LYs) due to halting the rise of overweight and obesity with the potential to earn higher income as follows:

Equation 1

$$pM=\sum_{i=1}^{n} LYiWA$$

Where *pM* is productivity changes due to reduced high BMI-related mortality, *LYi* is the number of added years lived by employed populations due to halted rise in overweight and obesity, and *WA* is the average annual wage rate in the working population in Kenya (US Dollars 655·05) [48]. In the model, we apply an adjustment for the percentage of the 2019 working population that is employed in Kenya (58·4%) [48]. Due to limitations in the data availability on sex- age specific average wage rate in Kenya, we apply one estimate across all age groups and sex. While this limits factual accuracy, it is recommended for promotion of equity in economic assessments [46].

Secondly, to estimate productivity gains resulting from a reduction in high BMI-related mortality and morbidity (combined), we use estimated HALYs gained by the working population in Kenya (age 20 to 65). We used the disability weight as a proxy for work-ability. Disability weights indicate the average loss of quality of life due to disease [49]. In the equation above *LYi* is the number of added years lived is replaced with HALY*i* which is the number of added health adjusted years lived in employed populations due to halted rise in overweight and obesity. Thirdly, we estimate productivity gains resulting from a reduction in high BMI-related morbidity. We use the same equation as above but in place of HALYs gained by the working population in Kenya, we use the high BMI-related morbidity calculated as the difference between HALYs gained and added years lived by employed populations (HALYs minus LYs).

# Additional results from main analysis

## 2.1 A 20% tax on sugar sweetened beverages

Table 14: Change in disease outcomes for the intervention, 20% tax on sugar sweetened beverages

| **Variable** | **Numbers of new cases averted (2019-2044)** | | | **Numbers of avoidable deaths ^#^ (2019-2044)** | | | **Numbers of avoidable prevalence cases (2044)** | | |
| --- | --- | --- | --- | --- | --- | --- | --- | --- | --- |
| **Sex specific mean and 95% UI** | **Female, n (95% UI)** | **Male, n (95% UI)** | **Total, n**  **(95% UI)** | **Female, n (95% UI)** | **Male, n (95% UI)** | **Total, n (95% UI)** | **Female, n (95% UI)** | **Male, n (95% UI)** | **Total, n**  **(95% UI)** |
| **High BMI related disease** |  |  |  |  |  |  |  |  |  |
| **Diabetes mellitus type 2** | 15,478 | 14,253 | 29,731 | 435 | 688 | 1,122 | 9,652 | 10,972 | 20,624 |
|  | (10,030 - 22,404) | (9,779 - 20,304) | (21,558 - 39,552) | (298 - 601) | (474 - 978) | (832 - 1,460) | (6,369 - 13,714) | (7,447 - 15,576) | (15,122 - 27,281) |
| **Cardiovascular diseases** |  |  |  |  |  |  |  |  |  |
| Ischemic heart disease | 1,779 | 2,190 | 3,969 | 302 | 372 | 675 | 1,125 | 1,343 | 2,467 |
|  | (1,081 - 2,751) | (1,391 - 3,222) | (2,813 - 5,388) | (181 - 460) | (235 - 550) | (476 - 914) | (649 - 1,811) | (822 - 2,035) | (1,684 - 3,443) |
| Ischemic stroke | 1,093 | 707 | 1,800 | 76 | 67 | 143 | 795 | 478 | 1,273 |
|  | (657 - 1,700) | (434 - 1,069) | (1,270 - 2,506) | (41 - 117) | (41 - 99) | (98 - 193) | (447 - 1,288) | (273 - 751) | (860 - 1,830) |
| Intracerebral haemorrhage | 1,692 | 1,545 | 3,237 | 697 | 659 | 1,356 | 860 | 731 | 1,591 |
|  | (876 - 2,837) | (875 - 2,500) | (2,068 - 4,739) | (381 - 1,120) | (385 - 1,030) | (894 - 1,941) | (419 - 1,501) | (395 - 1,228) | (982 - 2,413) |
| Subarachnoid haemorrhage | 329 | 273 | 602 | 48 | 56 | 104 | 167 | 126 | 293 |
|  | (172 - 550) | (145 - 452) | (384 - 885) | (26 - 79) | (30 - 91) | (67 - 151) | (81 - 293) | (62 - 218) | (177 - 447) |
| Hypertensive heart disease | 1,434 | 1,028 | 2,462 | 682 | 296 | 977 | 540 | 501 | 1,041 |
|  | (417 - 2,962) | (360 - 2,107) | (1,134 - 4,145) | (197 - 1,395) | (106 - 589) | (429 - 1,724) | (158 - 1,116) | (170 - 1,040) | (502 - 1,765) |
| Atrial fibrillation and flutter | 128 | 120 | 249 | 11 | 4 | 15 | 70 | 67 | 137 |
|  | (72 - 199) | (72 - 176) | (168 - 336) | (4 - 18) | (2 - 6) | (8 - 23) | (36 - 111) | (37 - 102) | (88 - 190) |
| **Cancers** |  |  |  |  |  |  |  |  |  |
| Oesophageal cancer | 110 | 91 | 201 | 95 | 78 | 173 | 10 | 9 | 19 |
|  | (1 - 268) | (13 - 194) | (65 - 382) | (0 - 231) | (10 - 166) | (55 - 329) | (0 - 26) | (1 - 19) | (6 - 38) |
| Colon cancer | 7 | 22 | 29 | 4 | 15 | 19 | 1 | 4 | 5 |
|  | (1 - 13) | (16 - 30) | (21 - 39) | (-1 - 9) | (11 - 20) | (13 - 26) | (0 - 2) | (3 - 6) | (3 - 7) |
| Liver cancer due to alcohol use | 2 | 5 | 7 | 2 | 5 | 6 | 0 | 0 | 0 |
|  | (0 - 4) | (1 - 10) | (3 - 12) | (0 - 3) | (1 - 9) | (3 - 11) | (0 - 0) | (0 - 1) | (0 - 1) |
| Liver cancer due to hepatitis B | 3 | 6 | 9 | 3 | 5 | 8 | 0 | 0 | 1 |
|  | (1 - 7) | (2 - 11) | (4 - 16) | (0 - 6) | (2 - 10) | (4 - 14) | (0 - 1) | (0 - 1) | (0 - 1) |
| Liver cancer due to hepatitis C | 3 | 2 | 5 | 2 | 2 | 4 | 0 | 0 | 0 |
|  | (0 - 6) | (1 - 4) | (2 - 9) | (0 - 5) | (1 - 4) | (2 - 8) | (0 - 0) | (0 - 0) | (0 - 1) |
| Gallbladder and biliary cancer | 15 | 2 | 16 | 13 | 2 | 14 | 1 | 0 | 1 |
|  | (8 - 23) | (0 - 4) | (10 - 25) | (7 - 20) | (0 - 3) | (8 - 22) | (1 - 2) | (0 - 0) | (1 - 2) |
| Pancreatic cancer | 5 | 2 | 7 | 4 | 2 | 6 | 0 | 0 | 0 |
|  | (1 - 10) | (-1 - 6) | (1 - 13) | (0 - 9) | (-1 - 5) | (1 - 12) | (0 - 0) | (0 - 0) | (0 - 1) |
| Breast cancer | 17 |  |  | 3 |  |  | 1 |  |  |
|  | (-8 - 44) |  |  | (-9 - 16) |  |  | (-7 - 11) |  |  |
| Uterine cancer | 45 |  |  | 20 |  |  | 15 |  |  |
|  | (35 - 56) |  |  | (15 - 25) |  |  | (12 - 19) |  |  |
| Ovarian cancer | 4 |  |  | 2 |  |  | 1 |  |  |
|  | (-3 - 11) |  |  | (-2 - 7) |  |  | (-1 - 3) |  |  |
| Kidney cancer | 8 | 4 | 12 | 5 | 2 | 7 | 2 | 1 | 3 |
|  | (6 - 11) | (3 - 6) | (9 - 15) | (3 - 6) | (2 - 3) | (5 - 9) | (1 - 2) | (1 - 1) | (2 - 3) |
| Thyroid cancer | 5 | 2 | 7 | 1 | 1 | 1 | 2 | 1 | 3 |
|  | (3 - 8) | (1 - 4) | (5 - 10) | (0 - 1) | (0 - 1) | (1 - 2) | (1 - 3) | (0 - 1) | (2 - 4) |
| Acute lymphoid leukaemia | 0 | 1 | 1 | 0 | 0 | 1 | 0 | 0 | 0 |
|  | (0 - 1) | (0 - 1) | (1 - 1) | (0 - 1) | (0 - 1) | (0 - 1) | (0 - 0) | (0 - 0) | (0 - 0) |
| Acute myeloid leukaemia | 1 | 1 | 3 | 1 | 1 | 2 | 0 | 0 | 0 |
|  | (1 - 3) | (1 - 2) | (2 - 4) | (0 - 2) | (0 - 1) | (1 - 3) | (0 - 0) | (0 - 0) | (0 - 0) |
| Chronic lymphoid leukaemia | 2 | 0 | 2 | 1 | 0 | 1 | 0 | 0 | 0 |
|  | (0 - 3) | (0 - 1) | (1 - 4) | (0 - 2) | (0 - 1) | (0 - 3) | (0 - 0) | (0 - 0) | (0 - 1) |
| Chronic myeloid leukaemia | 1 | 1 | 1 | 1 | 1 | 1 | 0 | 0 | 0 |
|  | (0 - 1) | (0 - 1) | (1 - 2) | (0 - 1) | (0 - 1) | (1 - 2) | (0 - 0) | (0 - 0) | (0 - 0) |
| Other Leukaemia | 1 | 2 | 3 | 1 | 1 | 2 | 0 | 0 | 1 |
|  | (0 - 2) | (1 - 3) | (2 - 4) | (0 - 1) | (0 - 2) | (1 - 2) | (0 - 1) | (0 - 1) | (0 - 1) |
| Multiple myeloma | 2 | 2 | 4 | 2 | 1 | 3 | 0 | 0 | 0 |
|  | (0 - 5) | (0 - 4) | (1 - 7) | (0 - 4) | (0 - 3) | (1 - 6) | (0 - 1) | (0 - 0) | (0 - 1) |
| **Chronic kidney disease** |  |  |  |  |  |  |  |  |  |
| CKD due to diabetes mellitus type 2 | 1,317 | 907 | 2,225 | 33 | 26 | 59 | 984 | 636 | 1,620 |
|  | (127 - 3,375) | (103 - 2,405) | (697 - 4,639) | (1 - 85) | (103 - 2,405) | (697 - 4,639) | (50 - 2,625) | (43 - 1,751) | (452 - 3,514) |
| CKD due to glomerulonephritis | 70 | 64 | 134 | 20 | 24 | 44 | 38 | 27 | 65 |
|  | (5 - 191) | (6 - 167) | (39 - 291) | (0 - 55) | (6 - 167) | (39 - 291) | (1 - 112) | (0 - 75) | (15 - 150) |
| CKD due to hypertension | 409 | 299 | 708 | 61 | 57 | 118 | 241 | 160 | 400 |
|  | (35 - 1,066) | (33 - 731) | (224 - 1,474) | (3 - 158) | (33 - 731) | (224 - 1,474) | (15 - 638) | (13 - 408) | (116 - 856) |
| CKD due to other and unspecified causes | 3,111 | 1,718 | 4,828 | 14 | 11 | 25 | 2,196 | 1,155 | 3,350 |
|  | (381 - 7,560) | (165 - 4,223) | (1,523 - 9,908) | (0 - 36) | (165 - 4,223) | (1,523 - 9,908) | (120 - 5,683) | (19 - 3,046) | (847 - 7,270) |
| **Musculoskeletal diseases** |  |  |  |  |  |  |  |  |  |
| Osteoarthritis hip | 59 | 60 | 119 |  |  |  | 32 | 32 | 64 |
|  | (29 - 94) | (30 - 95) | (75 - 169) |  |  |  | (7 - 60) | (8 - 58) | (29 - 105) |
| Osteoarthritis knee | 3,829 | 2,527 | 6,356 |  |  |  | 2,338 | 1,469 | 3,807 |
|  | (1,854 - 6,388) | (1,293 - 3,986) | (3,919 - 9,346) |  |  |  | (1,005 - 4,040) | (692 - 2,413) | (2,214 - 5,763) |
| Low back pain | 6,295 | 6,211 | 12,506 |  |  |  | 820 | 860 | 1,680 |
|  | (4,189 - 8,599) | (4,214 - 8,644) | (0 - 0) |  |  |  | (503 - 1,171) | (545 - 1,231) | (1,228 - 2,205) |
| Gout | 813 | 2,446 | 3,259 |  |  |  | 254 | 784 | 1,038 |
|  | (478 - 1,248) | (1,201 - 4,107) | (1,911 - 4,960) |  |  |  | (144 - 398) | (374 - 1,338) | (588 - 1,599) |
| **Other diseases** |  |  |  |  |  |  |  |  |  |
| Gallbladder and biliary diseases | 2,259 | 480 | 2,739 | 57 | 22 | 80 | 769 | 152 | 921 |
|  | (1,603 - 3,079) | (283 - 710) | (1,914 - 3,721) | (40 - 78) | (12 - 34) | (53 - 111) | (538 - 1,063) | (87 - 226) | (633 - 1,266) |
| Asthma | 2,439 | 1,552 | 3,991 | 58 | 32 | 89 | 1,119 | 688 | 1,807 |
|  | (1,529 - 3,563) | (971 - 2,258) | (2,536 - 5,693) | (35 - 85) | (19 - 47) | (55 - 129) | (686 - 1,652) | (423 - 1,015) | (1,130 - 2,596) |
| Alzheimer's disease and other dementias | 208 | 95 | 303 | 0 | 0 | 0 | 69 | 35 | 104 |
|  | (-6 - 489) | (9 - 201) | (-1 - 675) | (0 - 0) | (0 - 0) | (0 - 0) | (-46 - 216) | (-9 - 90) | (-55 - 300) |
| Cataract | 294 | 174 | 468 |  |  |  | 108 | 68 | 176 |
|  | (108 - 503) | (63 - 296) | (177 - 795) |  |  |  | (-31 - 269) | (-9 - 151) | (-36 - 414) |

UI: uncertainty interval, n: mean number ^#^Musculoskeletal diseases and cataract are not linked to disease specific mortality.

## 2.2 Mandatory kilojoule menu labelling

Table 15: Change in disease outcomes for the intervention, mandatory kilojoule menu labelling

| **Variable** | **Numbers of new cases averted (2019-2044)** | | | **Numbers of avoidable deaths^#^  (2019-2044)** | | | **Numbers of avoidable prevalence cases (2044)** | | |
| --- | --- | --- | --- | --- | --- | --- | --- | --- | --- |
| **Sex specific mean and 95% UI** | **Female, n (95% UI)** | **Male, n (95% UI)** | **Total, n (95% UI)** | **Female, n (95% UI)** | **Male, n (95% UI)** | **Total, n (95% UI)** | **Female, n (95% UI)** | **Male, n (95% UI)** | **Total, n**  **(95% UI)** |
| **High BMI related disease** |  |  |  |  |  |  |  |  |  |
| **Diabetes mellitus type 2** | 9,609 | 7,088 | 16,696 | 303 | 364 | 667 | 6,030 | 5,336 | 11,366 |
|  | (3,295 - 17,749) | (2,321 - 12,678) | (5,692 - 29,220) | (105 - 549) | (120 - 648) | (230 - 1,150) | (2,093 - 10,985) | (1,752 - 9,589) | (3,894 - 19,699) |
| **Cardiovascular diseases** |  |  |  |  |  |  |  |  |  |
| Ischemic heart disease | 1,578 | 1,611 | 3,189 | 285 | 280 | 564 | 930 | 931 | 1,861 |
|  | (523 - 2,926) | (521 - 2,858) | (1,089 - 5,599) | (93 - 529) | (89 - 497) | (192 - 990) | (298 - 1,763) | (295 - 1,687) | (630 - 3,311) |
| Ischemic stroke | 937 | 522 | 1,460 | 76 | 58 | 134 | 641 | 330 | 971 |
|  | (304 - 1,784) | (185 - 959) | (520 - 2,613) | (23 - 148) | (20 - 104) | (48 - 238) | (201 - 1,240) | (115 - 621) | (345 - 1,762) |
| Intracerebral haemorrhage | 1,418 | 1,092 | 2,510 | 626 | 495 | 1,121 | 673 | 481 | 1,154 |
|  | (452 - 2,849) | (374 - 2,131) | (861 - 4,619) | (203 - 1,231) | (169 - 951) | (387 - 2,032) | (208 - 1,381) | (161 - 968) | (390 - 2,166) |
| Subarachnoid haemorrhage | 273 | 189 | 462 | 41 | 40 | 81 | 130 | 81 | 210 |
|  | (83 - 549) | (55 - 365) | (153 - 851) | (13 - 82) | (12 - 76) | (27 - 147) | (37 - 272) | (23 - 161) | (68 - 399) |
| Hypertensive heart disease | 1,406 | 829 | 2,235 | 681 | 237 | 918 | 491 | 383 | 874 |
|  | (280 - 3,430) | (207 - 1,933) | (642 - 4,783) | (132 - 1,650) | (60 - 546) | (259 - 1,989) | (101 - 1,206) | (94 - 906) | (259 - 1,867) |
| Atrial fibrillation and flutter | 131 | 100 | 231 | 12 | 4 | 16 | 66 | 52 | 118 |
|  | (40 - 250) | (32 - 187) | (79 - 412) | (3 - 25) | (1 - 7) | (5 - 31) | (18 - 130) | (16 - 100) | (39 - 214) |
| **Cancers** |  |  |  |  |  |  |  |  |  |
| Oesophageal cancer | 110 | 75 | 186 | 96 | 65 | 160 | 10 | 7 | 16 |
|  | (0 - 302) | (12 - 189) | (39 - 413) | (-1 - 262) | (10 - 163) | (34 - 357) | (0 - 28) | (1 - 17) | (3 - 38) |
| Colon cancer | 6 | 18 | 25 | 3 | 13 | 16 | 1 | 3 | 4 |
|  | (0 - 15) | (6 - 32) | (8 - 44) | (-1 - 10) | (4 - 22) | (5 - 29) | (0 - 2) | (1 - 5) | (1 - 7) |
| Liver cancer due to alcohol use | 2 | 5 | 6 | 2 | 4 | 6 | 0 | 0 | 0 |
|  | (0 - 4) | (1 - 10) | (2 - 13) | (0 - 4) | (1 - 9) | (1 - 12) | (0 - 0) | (0 - 1) | (0 - 1) |
| Liver cancer due to hepatitis B | 3 | 5 | 8 | 3 | 4 | 7 | 0 | 0 | 1 |
|  | (0 - 7) | (1 - 10) | (2 - 16) | (0 - 7) | (1 - 9) | (2 - 14) | (0 - 0) | (0 - 1) | (0 - 1) |
| Liver cancer due to hepatitis C | 3 | 2 | 5 | 2 | 2 | 4 | 0 | 0 | 0 |
|  | (0 - 7) | (0 - 5) | (1 - 10) | (0 - 6) | (0 - 4) | (1 - 9) | (0 - 0) | (0 - 0) | (0 - 1) |
| Gallbladder and biliary cancer | 15 | 1 | 16 | 13 | 1 | 14 | 1 | 0 | 1 |
|  | (5 - 28) | (0 - 4) | (5 - 30) | (4 - 25) | (0 - 3) | (5 - 27) | (0 - 2) | (0 - 0) | (0 - 2) |
| Pancreatic cancer | 5 | 2 | 7 | 4 | 2 | 6 | 0 | 0 | 0 |
|  | (0 - 12) | (-1 - 6) | (1 - 15) | (0 - 11) | (-1 - 5) | (1 - 14) | (0 - 0) | (0 - 0) | (0 - 1) |
| Breast cancer | 20 |  |  | 5 |  |  | 2 |  |  |
|  | (-5 - 53) |  |  | (-8 - 20) |  |  | (-6 - 11) |  |  |
| Uterine cancer | 43 |  |  | 20 |  |  | 13 |  |  |
|  | (15 - 73) |  |  | (7 - 33) |  |  | (5 - 22) |  |  |
| Ovarian cancer | 3 |  |  | 2 |  |  | 1 |  |  |
|  | (-2 - 11) |  |  | (-3 - 7) |  |  | (-1 - 2) |  |  |
| Kidney cancer | 8 | 3 | 11 | 5 | 2 | 7 | 2 | 1 | 2 |
|  | (3 - 14) | (1 - 6) | (4 - 19) | (2 - 8) | (1 - 4) | (2 - 11) | (1 - 3) | (0 - 1) | (1 - 4) |
| Thyroid cancer | 4 | 2 | 6 | 1 | 0 | 1 | 1 | 0 | 2 |
|  | (1 - 8) | (0 - 3) | (2 - 11) | (0 - 1) | (0 - 1) | (0 - 2) | (0 - 3) | (0 - 1) | (1 - 3) |
| Acute lymphoid leukaemia | 0 | 0 | 1 | 0 | 0 | 1 | 0 | 0 | 0 |
|  | (0 - 1) | (0 - 1) | (0 - 1) | (0 - 1) | (0 - 1) | (0 - 1) | (0 - 0) | (0 - 0) | (0 - 0) |
| Acute myeloid leukaemia | 1 | 1 | 2 | 1 | 1 | 2 | 0 | 0 | 0 |
|  | (0 - 2) | (0 - 2) | (1 - 4) | (0 - 2) | (0 - 1) | (0 - 3) | (0 - 0) | (0 - 0) | (0 - 0) |
| Chronic lymphoid leukaemia | 2 | 0 | 2 | 1 | 0 | 1 | 0 | 0 | 0 |
|  | (0 - 4) | (0 - 1) | (0 - 4) | (0 - 3) | (0 - 1) | (0 - 3) | (0 - 1) | (0 - 0) | (0 - 1) |
| Chronic myeloid leukaemia | 1 | 0 | 1 | 0 | 0 | 1 | 0 | 0 | 0 |
|  | (0 - 1) | (0 - 1) | (0 - 2) | (0 - 1) | (0 - 1) | (0 - 1) | (0 - 0) | (0 - 0) | (0 - 0) |
| Other Leukaemia | 1 | 1 | 2 | 1 | 1 | 1 | 0 | 0 | 1 |
|  | (0 - 2) | (0 - 3) | (1 - 4) | (0 - 1) | (0 - 2) | (0 - 2) | (0 - 1) | (0 - 1) | (0 - 1) |
| Multiple myeloma | 2 | 1 | 4 | 2 | 1 | 3 | 0 | 0 | 0 |
|  | (0 - 5) | (0 - 4) | (1 - 8) | (0 - 4) | (0 - 3) | (0 - 6) | (0 - 1) | (0 - 0) | (0 - 1) |
| **Chronic kidney disease** |  |  |  |  |  |  |  |  |  |
| CKD due to diabetes mellitus type 2 | 1,176 | 663 | 1,840 | 34 | 22 | 56 | 840 | 442 | 1,282 |
|  | (80 - 3,277) | (63 - 1,846) | (365 - 4,362) | (0 - 98) | (63 - 1,846) | (365 - 4,362) | (28 - 2,430) | (17 - 1,275) | (224 - 3,164) |
| CKD due to glomerulonephritis | 64 | 54 | 119 | 21 | 22 | 43 | 31 | 20 | 52 |
|  | (3 - 197) | (4 - 157) | (21 - 294) | (0 - 66) | (4 - 157) | (21 - 294) | (0 - 100) | (1 - 64) | (8 - 135) |
| CKD due to hypertension | 393 | 257 | 650 | 64 | 52 | 115 | 215 | 127 | 342 |
|  | (21 - 1,149) | (23 - 701) | (124 - 1,574) | (1 - 188) | (23 - 701) | (124 - 1,574) | (8 - 642) | (9 - 361) | (62 - 848) |
| CKD due to other and unspecified causes | 2,935 | 1,369 | 4,303 | 15 | 10 | 25 | 1,963 | 871 | 2,834 |
|  | (256 - 8,721) | (127 - 4,069) | (808 - 10,509) | (0 - 47) | (127 - 4,069) | (808 - 10,509) | (47 - 6,202) | (12 - 2,787) | (365 - 7,370) |
| **Musculoskeletal diseases** |  |  |  |  |  |  |  |  |  |
| Osteoarthritis hip | 50 | 44 | 95 |  |  |  | 24 | 21 | 45 |
|  | (15 - 98) | (13 - 87) | (33 - 170) |  |  |  | (2 - 56) | (3 - 48) | (12 - 90) |
| Osteoarthritis knee | 3,382 | 1,860 | 5,242 |  |  |  | 1,984 | 1,039 | 3,023 |
|  | (975 - 6,876) | (563 - 3,652) | (1,675 - 9,865) |  |  |  | (507 - 4,138) | (302 - 2,080) | (917 - 5,828) |
| Low back pain | 5,176 | 4,228 | 9,404 |  |  |  | 634 | 558 | 1,193 |
|  | (1,741 - 9,194) | (1,447 - 7,558) | (0 - 0) |  |  |  | (206 - 1,178) | (192 - 1,019) | (415 - 2,077) |
| Gout | 733 | 1,831 | 2,564 |  |  |  | 217 | 560 | 777 |
|  | (242 - 1,373) | (546 - 3,658) | (857 - 4,766) |  |  |  | (69 - 415) | (165 - 1,132) | (256 - 1,462) |
| **Other diseases** |  |  |  |  |  |  |  |  |  |
| Gallbladder and biliary diseases | 1,891 | 342 | 2,233 | 60 | 20 | 80 | 616 | 103 | 718 |
|  | (673 - 3,278) | (119 - 638) | (799 - 3,898) | (21 - 106) | (7 - 37) | (29 - 143) | (219 - 1,072) | (35 - 192) | (258 - 1,266) |
| Asthma | 1,944 | 1,080 | 3,024 | 55 | 26 | 80 | 858 | 459 | 1,317 |
|  | (638 - 3,625) | (362 - 1,999) | (1,000 - 5,648) | (17 - 105) | (8 - 48) | (26 - 153) | (278 - 1,619) | (152 - 856) | (430 - 2,471) |
| Alzheimer's disease and other dementias | 223 | 85 | 308 | 0 | 0 | 0 | 64 | 28 | 93 |
|  | (-21 - 616) | (0 - 219) | (-18 - 835) | (0 - 0) | (0 - 0) | (0 - 0) | (-62 - 248) | (-12 - 89) | (-72 - 336) |
| Cataract | 272 | 137 | 408 |  |  |  | 73 | 43 | 116 |
|  | (53 - 586) | (27 - 287) | (80 - 866) |  |  |  | (-70 - 250) | (-18 - 127) | (-86 - 379) |

UI: uncertainty interval, n: mean number ^#^Musculoskeletal diseases and cataract are not linked to disease specific mortality.

## 2.3 Change in consumption levels related to supermarket food purchase

Table 16: Change in disease outcomes for the intervention, change in consumption levels related to supermarket food purchase

| **Variable** | **Numbers of new cases averted (2019-2044)** | | | **Numbers of avoidable deaths^#^ (2019-2044)** | | | **Numbers of avoidable prevalence cases (2044)** | | |
| --- | --- | --- | --- | --- | --- | --- | --- | --- | --- |
| **Sex specific mean and 95% UI** | **Female, n (95% UI)** | **Male, n**  **(95% UI)** | **Total, n**  **(95% UI)** | **Female, n (95% UI)** | **Male, n**  **(95% UI)** | **Total, n**  **(95% UI)** | **Female, n (95% UI)** | **Male, n**  **(95% UI)** | **Total, n**  **(95% UI)** |
| **High BMI related disease** |  |  |  |  |  |  |  |  |  |
| **Diabetes mellitus type 2** | 193,102 | 157,340 | 350,442 | 4,224 | 5,565 | 9,788 | 131,776 | 127,293 | 259,069 |
|  | (119,703 - 280,238) | (100,805 - 228,190) | (239,273 - 473,272) | (2,631 - 6,018) | (3,587 - 8,022) | (6,671 - 13,245) | (83,016 - 187,072) | (81,280 - 185,173) | (176,932 - 348,568) |
| **Cardiovascular diseases** |  |  |  |  |  |  |  |  |  |
| Ischemic heart disease | 33,434 | 37,947 | 71,381 | 4,653 | 5,040 | 9,694 | 22,340 | 25,309 | 47,649 |
|  | (20,366 - 51,985) | (23,722 - 55,942) | (47,550 - 99,921) | (2,711 - 7,151) | (3,149 - 7,458) | (6,401 - 13,582) | (13,203 - 35,429) | (15,480 - 38,374) | (31,308 - 67,874) |
| Ischemic stroke | 19,934 | 12,010 | 31,944 | 1,207 | 1,002 | 2,209 | 14,867 | 8,507 | 23,374 |
|  | (12,107 - 31,310) | (7,706 - 17,356) | (21,509 - 45,186) | (653 - 1,954) | (645 - 1,424) | (1,468 - 3,114) | (8,785 - 24,006) | (5,327 - 12,501) | (15,454 - 33,793) |
| Intracerebral haemorrhage | 29,032 | 24,620 | 53,652 | 10,549 | 9,173 | 19,722 | 16,224 | 13,120 | 29,344 |
|  | (14,993 - 47,408) | (13,035 - 39,437) | (32,812 - 78,914) | (5,650 - 16,866) | (4,980 - 14,376) | (12,211 - 28,596) | (8,133 - 26,904) | (6,782 - 21,475) | (17,582 - 44,152) |
| Subarachnoid haemorrhage | 5,555 | 4,256 | 9,811 | 692 | 746 | 1,438 | 3,113 | 2,206 | 5,319 |
|  | (2,910 - 9,305) | (2,259 - 6,721) | (5,938 - 14,704) | (370 - 1,140) | (399 - 1,164) | (890 - 2,094) | (1,554 - 5,381) | (1,125 - 3,582) | (3,115 - 8,146) |
| Hypertensive heart disease | 29,901 | 18,698 | 48,600 | 12,612 | 4,374 | 16,985 | 12,836 | 10,399 | 23,235 |
|  | (8,398 - 65,488) | (6,509 - 37,476) | (20,976 - 90,524) | (3,422 - 27,670) | (1,543 - 8,648) | (6,936 - 32,935) | (3,791 - 27,843) | (3,592 - 20,914) | (10,107 - 42,381) |
| Atrial fibrillation and flutter | 2,794 | 2,356 | 5,150 | 213 | 69 | 281 | 1,665 | 1,458 | 3,122 |
|  | (1,529 - 4,430) | (1,369 - 3,593) | (3,324 - 7,244) | (93 - 364) | (37 - 109) | (154 - 442) | (840 - 2,723) | (795 - 2,293) | (1,937 - 4,500) |
| **Cancers** |  |  |  |  |  |  |  |  |  |
| Oesophageal cancer | 2,399 | 1,720 | 4,119 | 2,016 | 1,430 | 3,445 | 278 | 208 | 486 |
|  | (84 - 5,896) | (299 - 3,544) | (1,226 - 7,960) | (58 - 4,953) | (244 - 2,946) | (1,018 - 6,666) | (3 - 707) | (32 - 438) | (136 - 961) |
| Colon cancer | 179 | 450 | 629 | 104 | 287 | 391 | 33 | 106 | 139 |
|  | (57 - 318) | (317 - 599) | (425 - 864) | (20 - 197) | (201 - 384) | (258 - 545) | (3 - 66) | (73 - 143) | (90 - 196) |
| Liver cancer due to alcohol use | 39 | 109 | 148 | 35 | 94 | 129 | 3 | 9 | 12 |
|  | (5 - 84) | (34 - 200) | (63 - 255) | (4 - 74) | (29 - 174) | (55 - 223) | (0 - 7) | (3 - 18) | (5 - 22) |
| Liver cancer due to hepatitis B | 63 | 110 | 173 | 56 | 95 | 151 | 6 | 10 | 16 |
|  | (9 - 135) | (36 - 205) | (74 - 294) | (8 - 119) | (31 - 178) | (65 - 258) | (1 - 12) | (3 - 19) | (6 - 27) |
| Liver cancer due to hepatitis C | 63 | 49 | 112 | 55 | 42 | 97 | 5 | 4 | 9 |
|  | (5 - 137) | (16 - 92) | (43 - 197) | (4 - 120) | (14 - 80) | (37 - 173) | (0 - 11) | (1 - 8) | (3 - 16) |
| Gallbladder and biliary cancer | 320 | 37 | 357 | 277 | 31 | 308 | 29 | 3 | 32 |
|  | (173 - 498) | (3 - 75) | (202 - 551) | (149 - 431) | (2 - 63) | (174 - 476) | (15 - 45) | (0 - 7) | (17 - 50) |
| Pancreatic cancer | 124 | 51 | 175 | 111 | 45 | 156 | 7 | 3 | 10 |
|  | (31 - 240) | (-15 - 124) | (55 - 315) | (26 - 216) | (-15 - 110) | (47 - 283) | (1 - 14) | (-2 - 8) | (2 - 19) |
| Breast cancer | 513 |  | 513 | 149 |  | 149 | 120 |  | 120 |
|  | (5 - 1,058) |  | (5 - 1,058) | (-72 - 388) |  | (-72 - 388) | (-91 - 351) |  | (-91 - 351) |
| Uterine cancer | 896 |  | 896 | 362 |  | 362 | 342 |  | 342 |
|  | (640 - 1,152) |  | (640 - 1,152) | (259 - 464) |  | (259 - 464) | (244 - 440) |  | (244 - 440) |
| Ovarian cancer | 93 |  | 93 | 45 |  | 45 | 23 |  | 23 |
|  | (-29 - 235) |  | (-29 - 235) | (-30 - 131) |  | (-30 - 131) | (-13 - 64) |  | (-13 - 64) |
| Kidney cancer | 164 | 77 | 241 | 91 | 43 | 134 | 41 | 20 | 61 |
|  | (110 - 230) | (48 - 110) | (168 - 327) | (61 - 128) | (26 - 62) | (93 - 182) | (28 - 58) | (12 - 29) | (43 - 83) |
| Thyroid cancer | 89 | 36 | 124 | 15 | 10 | 25 | 39 | 14 | 52 |
|  | (55 - 130) | (10 - 68) | (79 - 178) | (9 - 22) | (3 - 20) | (15 - 38) | (24 - 57) | (4 - 26) | (33 - 75) |
| Acute lymphoid leukaemia | 7 | 9 | 16 | 5 | 7 | 12 | 1 | 1 | 2 |
|  | (3 - 12) | (5 - 14) | (10 - 24) | (2 - 9) | (4 - 10) | (7 - 17) | (0 - 2) | (1 - 2) | (1 - 3) |
| Acute myeloid leukaemia | 25 | 20 | 44 | 20 | 15 | 35 | 4 | 2 | 6 |
|  | (10 - 42) | (11 - 30) | (25 - 66) | (8 - 34) | (8 - 23) | (20 - 53) | (1 - 7) | (1 - 3) | (3 - 9) |
| Chronic lymphoid leukaemia | 40 | 11 | 51 | 27 | 7 | 34 | 7 | 2 | 9 |
|  | (12 - 72) | (5 - 17) | (22 - 85) | (7 - 50) | (3 - 11) | (14 - 58) | (2 - 14) | (1 - 4) | (4 - 16) |
| Chronic myeloid leukaemia | 11 | 11 | 22 | 9 | 9 | 18 | 1 | 1 | 2 |
|  | (4 - 18) | (6 - 18) | (13 - 33) | (3 - 15) | (5 - 15) | (11 - 28) | (0 - 2) | (1 - 2) | (1 - 4) |
| Other Leukaemia | 20 | 36 | 57 | 10 | 18 | 29 | 7 | 11 | 18 |
|  | (8 - 35) | (19 - 57) | (33 - 84) | (4 - 18) | (9 - 29) | (16 - 43) | (3 - 12) | (5 - 17) | (10 - 27) |
| Multiple myeloma | 55 | 39 | 94 | 41 | 29 | 70 | 8 | 6 | 13 |
|  | (9 - 107) | (7 - 75) | (38 - 160) | (5 - 83) | (4 - 56) | (25 - 122) | (0 - 16) | (0 - 12) | (4 - 24) |
| **Chronic kidney disease** |  |  |  |  |  |  |  |  |  |
| CKD due to diabetes mellitus type 2 | 24,201 | 15,027 | 39,228 | 493 | 350 | 842 | 18,738 | 11,151 | 29,889 |
|  | (1,754 - 66,848) | (1,814 - 38,926) | (11,746 - 83,778) | (-8 - 1,375) | (1,814 - 38,926) | (11,746 - 83,778) | (481 - 53,384) | (831 - 29,915) | (8,012 - 66,406) |
| CKD due to glomerulonephritis | 1,343 | 1,242 | 2,585 | 376 | 434 | 811 | 748 | 575 | 1,323 |
|  | (93 - 3,592) | (119 - 3,099) | (702 - 5,487) | (5 - 1,023) | (119 - 3,099) | (702 - 5,487) | (17 - 2,065) | (24 - 1,522) | (312 - 2,951) |
| CKD due to hypertension | 8,477 | 5,627 | 14,104 | 1,084 | 903 | 1,987 | 5,350 | 3,325 | 8,674 |
|  | (957 - 22,002) | (488 - 13,593) | (4,190 - 29,344) | (75 - 2,848) | (488 - 13,593) | (4,190 - 29,344) | (538 - 13,956) | (251 - 8,205) | (2,443 - 18,383) |
| CKD due to other and unspecified causes | 59,919 | 31,205 | 91,123 | 222 | 165 | 386 | 44,070 | 22,332 | 66,403 |
|  | (6,081 - 154,657) | (3,913 - 78,191) | (29,124 - 195,048) | (-6 - 603) | (3,913 - 78,191) | (29,124 - 195,048) | (1,019 - 118,995) | (1,514 - 58,843) | (17,858 - 147,864) |
| **Musculoskeletal diseases** |  |  |  |  |  |  |  |  |  |
| Osteoarthritis hip | 1,097 | 1,068 | 2,165 |  |  |  | 625 | 626 | 1,251 |
|  | (548 - 1,766) | (523 - 1,705) | (1,307 - 3,187) |  |  |  | (173 - 1,166) | (191 - 1,127) | (577 - 2,029) |
| Osteoarthritis knee | 72,196 | 43,133 | 115,329 |  |  |  | 48,526 | 28,209 | 76,735 |
|  | (33,786 - 120,111) | (22,046 - 67,775) | (66,629 - 172,748) |  |  |  | (20,954 - 82,803) | (13,684 - 45,323) | (42,305 - 117,241) |
| Low back pain | 112,651 | 101,329 | 213,980 |  |  |  | 20,358 | 19,293 | 39,651 |
|  | (72,487 - 159,431) | (65,446 - 141,185) | (0 - 3) |  |  |  | (12,319 - 29,821) | (12,038 - 27,444) | (26,592 - 53,544) |
| Gout | 15,110 | 41,605 | 56,716 |  |  |  | 5,797 | 16,493 | 22,290 |
|  | (8,515 - 23,242) | (20,910 - 70,485) | (33,057 - 87,857) |  |  |  | (3,203 - 9,016) | (8,145 - 28,310) | (12,705 - 35,017) |
| **Other diseases** |  |  |  |  |  |  |  |  |  |
| Gallbladder and biliary diseases | 38,667 | 7,738 | 46,405 | 1,077 | 396 | 1,474 | 16,005 | 3,019 | 19,024 |
|  | (26,027 - 53,582) | (4,563 - 11,710) | (30,467 - 65,276) | (713 - 1,508) | (232 - 603) | (941 - 2,107) | (10,664 - 22,305) | (1,761 - 4,599) | (12,397 - 26,918) |
| Asthma | 40,015 | 24,617 | 64,632 | 903 | 470 | 1,373 | 21,529 | 12,889 | 34,419 |
|  | (24,237 - 59,949) | (15,428 - 35,926) | (39,678 - 95,911) | (527 - 1,374) | (288 - 693) | (814 - 2,067) | (12,826 - 32,476) | (7,960 - 18,970) | (20,818 - 51,525) |
| Alzheimer's disease and other dementias | 5,483 | 2,225 | 7,708 | 0 | 0 | 0 | 2,324 | 1,031 | 3,355 |
|  | (563 - 12,038) | (373 - 4,528) | (943 - 16,618) | (0 - 0) | (0 - 0) | (0 - 0) | (-604 - 6,160) | (-37 - 2,353) | (-561 - 8,580) |
| Cataract | 6,391 | 3,571 | 9,962 |  |  |  | 2,614 | 1,671 | 4,285 |
|  | (2,499 - 10,784) | (1,525 - 5,964) | (4,141 - 16,755) |  |  |  | (-383 - 6,010) | (157 - 3,394) | (-170 - 9,401) |

UI: uncertainty interval, n: mean number ^#^Musculoskeletal diseases and cataract are not linked to disease specific mortality.

## 2.4 Change in national consumption levels back to the 1975 average level of energy intake

Table 17: Change in disease outcomes for the intervention, change in national consumption levels back to the 1975 average levels of energy intake

| **Variable** | **Numbers of new cases averted (2019-2044)** | | | **Numbers of avoidable deaths^#^ (2019-2044)** | | | **Numbers of avoidable prevalence cases (2044)** | | |
| --- | --- | --- | --- | --- | --- | --- | --- | --- | --- |
| **Sex specific mean and 95% UI** | **Female, n (95% UI)** | **Male, n (95% UI)** | **Total, n (95% UI)** | **Female, n (95% UI)** | **Male, n (95% UI)** | **Total, n (95% UI)** | **Female, n (95% UI)** | **Male, n (95% UI)** | **Total, n (95% UI)** |
| **High BMI related disease** |  |  |  |  |  |  |  |  |  |
| **Diabetes mellitus type 2** | 611,983 | 430,904 | 1,042,887 | 14,301 | 15,497 | 29,798 | 425,913 | 347,588 | 773,500 |
|  | (441,380 - 791,769) | (319,726 - 554,662) | (827,863 - 1,264,903) | (10,255 - 18,492) | (11,509 - 19,876) | (23,738 - 36,059) | (314,337 - 544,234) | (257,207 - 448,409) | (620,520 - 931,184) |
| **Cardiovascular diseases** |  |  |  |  |  |  |  |  |  |
| Ischemic heart disease | 135,633 | 119,915 | 255,548 | 19,304 | 15,797 | 35,100 | 90,552 | 80,637 | 171,189 |
|  | (95,834 - 183,421) | (86,110 - 156,665) | (198,794 - 315,146) | (13,220 - 26,375) | (11,238 - 20,677) | (26,941 - 43,457) | (62,619 - 124,830) | (56,447 - 107,080) | (130,588 - 213,280) |
| Ischemic stroke | 77,203 | 37,204 | 114,407 | 5,120 | 3,134 | 8,254 | 57,071 | 26,421 | 83,492 |
|  | (53,251 - 104,619) | (26,691 - 49,347) | (87,463 - 145,372) | (3,057 - 7,243) | (2,231 - 4,113) | (5,945 - 10,765) | (38,674 - 78,595) | (18,523 - 35,742) | (62,741 - 107,961) |
| Intracerebral haemorrhage | 103,271 | 72,592 | 175,863 | 38,757 | 27,278 | 66,035 | 56,401 | 38,438 | 94,839 |
|  | (63,855 - 148,071) | (44,607 - 104,394) | (125,132 - 230,164) | (24,117 - 54,798) | (16,892 - 38,553) | (47,433 - 85,635) | (34,413 - 81,929) | (23,344 - 56,089) | (66,648 - 125,404) |
| Subarachnoid haemorrhage | 19,370 | 12,454 | 31,824 | 2,448 | 2,184 | 4,632 | 10,605 | 6,379 | 16,984 |
|  | (11,924 - 28,249) | (7,596 - 17,974) | (22,578 - 42,865) | (1,507 - 3,522) | (1,323 - 3,128) | (3,300 - 6,121) | (6,316 - 15,835) | (3,770 - 9,435) | (11,751 - 23,225) |
| Hypertensive heart disease | 107,629 | 52,759 | 160,387 | 45,134 | 12,225 | 57,360 | 46,313 | 29,601 | 75,914 |
|  | (36,840 - 203,372) | (19,985 - 91,473) | (78,812 - 263,394) | (14,989 - 85,333) | (4,706 - 20,924) | (24,950 - 97,950) | (16,611 - 86,832) | (11,226 - 51,435) | (38,851 - 123,911) |
| Atrial fibrillation and flutter | 11,811 | 7,613 | 19,424 | 917 | 217 | 1,134 | 7,196 | 4,843 | 12,039 |
|  | (7,430 - 16,717) | (4,848 - 10,356) | (14,115 - 24,856) | (492 - 1,387) | (128 - 305) | (708 - 1,620) | (4,232 - 10,465) | (2,916 - 6,765) | (8,458 - 15,703) |
| **Cancers** |  |  |  |  |  |  |  |  |  |
| Oesophageal cancer | 10,009 | 5,471 | 15,480 | 8,397 | 4,532 | 12,929 | 1,190 | 685 | 1,874 |
|  | (394 - 22,754) | (1,129 - 10,704) | (4,485 - 29,309) | (284 - 19,073) | (922 - 8,870) | (3,691 - 24,493) | (19 - 2,806) | (127 - 1,371) | (530 - 3,631) |
| Colon cancer | 885 | 1,495 | 2,379 | 534 | 948 | 1,482 | 178 | 366 | 544 |
|  | (384 - 1,394) | (1,212 - 1,790) | (1,810 - 2,977) | (188 - 886) | (764 - 1,142) | (1,095 - 1,881) | (51 - 307) | (291 - 443) | (399 - 694) |
| Liver cancer due to alcohol use | 169 | 344 | 513 | 148 | 298 | 447 | 14 | 31 | 45 |
|  | (14 - 348) | (114 - 596) | (230 - 810) | (11 - 307) | (98 - 517) | (200 - 706) | (0 - 30) | (9 - 54) | (19 - 72) |
| Liver cancer due to hepatitis B | 273 | 365 | 638 | 240 | 317 | 557 | 25 | 35 | 60 |
|  | (38 - 540) | (134 - 625) | (303 - 1,020) | (32 - 476) | (115 - 543) | (263 - 890) | (3 - 51) | (12 - 61) | (27 - 97) |
| Liver cancer due to hepatitis C | 273 | 158 | 431 | 239 | 136 | 375 | 21 | 13 | 35 |
|  | (25 - 556) | (56 - 276) | (161 - 742) | (20 - 488) | (48 - 239) | (138 - 649) | (0 - 46) | (4 - 24) | (12 - 62) |
| Gallbladder and biliary cancer | 1,346 | 122 | 1,469 | 1,165 | 102 | 1,267 | 124 | 11 | 136 |
|  | (831 - 1,898) | (20 - 240) | (934 - 2,028) | (718 - 1,642) | (15 - 202) | (804 - 1,752) | (74 - 178) | (1 - 24) | (84 - 191) |
| Pancreatic cancer | 579 | 176 | 754 | 519 | 155 | 674 | 34 | 11 | 44 |
|  | (188 - 991) | (-42 - 414) | (290 - 1,232) | (164 - 894) | (-41 - 370) | (253 - 1,106) | (8 - 62) | (-5 - 28) | (13 - 77) |
| Breast cancer | 1,944 |  |  | 514 |  |  | 440 |  |  |
|  | (-326 - 4,348) |  |  | (-467 - 1,550) |  |  | (-521 - 1,459) |  |  |
| Uterine cancer | 3,548 |  |  | 1,431 |  |  | 1,368 |  |  |
|  | (3,106 - 3,995) |  |  | (1,251 - 1,610) |  |  | (1,194 - 1,544) |  |  |
| Ovarian cancer | 440 |  |  | 225 |  |  | 114 |  |  |
|  | (-129 - 1,032) |  |  | (-125 - 586) |  |  | (-55 - 290) |  |  |
| Kidney cancer | 694 | 255 | 949 | 385 | 140 | 525 | 176 | 69 | 245 |
|  | (537 - 860) | (180 - 338) | (772 - 1,139) | (297 - 477) | (98 - 187) | (427 - 633) | (136 - 219) | (48 - 92) | (199 - 296) |
| Thyroid cancer | 387 | 117 | 505 | 67 | 34 | 100 | 169 | 46 | 214 |
|  | (268 - 509) | (41 - 205) | (362 - 657) | (44 - 90) | (11 - 60) | (68 - 136) | (116 - 223) | (15 - 80) | (152 - 281) |
| Acute lymphoid leukaemia | 31 | 32 | 62 | 22 | 23 | 45 | 4 | 4 | 8 |
|  | (14 - 50) | (20 - 44) | (41 - 85) | (10 - 35) | (14 - 33) | (30 - 62) | (2 - 6) | (2 - 6) | (5 - 11) |
| Acute myeloid leukaemia | 107 | 66 | 173 | 87 | 50 | 137 | 17 | 6 | 23 |
|  | (46 - 171) | (39 - 95) | (107 - 243) | (37 - 140) | (29 - 72) | (83 - 194) | (7 - 27) | (3 - 9) | (13 - 33) |
| Chronic lymphoid leukaemia | 183 | 38 | 221 | 124 | 24 | 148 | 34 | 8 | 42 |
|  | (62 - 304) | (21 - 56) | (99 - 341) | (37 - 211) | (12 - 36) | (59 - 235) | (9 - 60) | (4 - 12) | (16 - 68) |
| Chronic myeloid leukaemia | 46 | 39 | 85 | 39 | 32 | 71 | 5 | 4 | 9 |
|  | (20 - 74) | (22 - 55) | (54 - 117) | (17 - 62) | (18 - 46) | (44 - 98) | (2 - 8) | (2 - 6) | (6 - 13) |
| Other Leukaemia | 87 | 125 | 212 | 44 | 63 | 108 | 31 | 39 | 69 |
|  | (39 - 138) | (72 - 180) | (141 - 289) | (19 - 70) | (33 - 93) | (68 - 149) | (13 - 49) | (20 - 58) | (44 - 96) |
| Multiple myeloma | 258 | 130 | 388 | 195 | 96 | 291 | 39 | 20 | 59 |
|  | (52 - 465) | (23 - 240) | (159 - 630) | (33 - 360) | (14 - 181) | (111 - 482) | (5 - 73) | (1 - 39) | (20 - 99) |
| **Chronic kidney disease** |  |  |  |  |  |  |  |  |  |
| CKD due to diabetes mellitus type 2 | 88,367 | 45,275 | 133,642 | 1,796 | 1,017 | 2,813 | 68,609 | 33,929 | 102,537 |
|  | (11,698 - 206,020) | (6,798 - 96,833) | (44,234 - 259,280) | (55 - 4,252) | (6,798 - 96,833) | (44,234 - 259,280) | (6,172 - 164,487) | (3,516 - 74,881) | (30,302 - 204,988) |
| CKD due to glomerulonephritis | 4,969 | 3,604 | 8,574 | 1,413 | 1,230 | 2,643 | 2,764 | 1,705 | 4,469 |
|  | (382 - 11,637) | (380 - 8,291) | (2,598 - 16,541) | (38 - 3,393) | (380 - 8,291) | (2,598 - 16,541) | (138 - 6,614) | (115 - 4,141) | (1,182 - 8,990) |
| CKD due to hypertension | 30,545 | 16,894 | 47,439 | 3,904 | 2,660 | 6,563 | 19,354 | 10,157 | 29,511 |
|  | (2,734 - 69,717) | (2,279 - 36,652) | (14,665 - 89,931) | (149 - 9,026) | (2,279 - 36,652) | (14,665 - 89,931) | (1,671 - 44,299) | (1,352 - 22,423) | (8,795 - 56,430) |
| CKD due to other and unspecified causes | 235,555 | 94,120 | 329,674 | 877 | 479 | 1,356 | 174,586 | 68,177 | 242,763 |
|  | (36,400 - 535,840) | (8,718 - 207,491) | (108,844 - 646,132) | (30 - 2,085) | (8,718 - 207,491) | (108,844 - 646,132) | (16,436 - 412,297) | (1,976 - 157,228) | (69,546 - 492,678) |
| **Musculoskeletal diseases** |  |  |  |  |  |  |  |  |  |
| Osteoarthritis hip | 4,858 | 3,644 | 8,502 |  |  |  | 2,955 | 2,254 | 5,210 |
|  | (2,581 - 7,245) | (2,045 - 5,339) | (5,706 - 11,357) |  |  |  | (955 - 4,981) | (929 - 3,653) | (2,809 - 7,648) |
| Osteoarthritis knee | 298,990 | 141,197 | 440,187 |  |  |  | 204,071 | 94,076 | 298,147 |
|  | (164,846 - 457,458) | (79,907 - 212,955) | (288,730 - 618,618) |  |  |  | (106,447 - 319,392) | (50,579 - 145,144) | (188,484 - 426,944) |
| Low back pain | 506,816 | 345,431 | 852,248 |  |  |  | 96,093 | 68,166 | 164,258 |
|  | (371,571 - 655,284) | (254,853 - 437,767) | (1 - 13) |  |  |  | (67,193 - 127,469) | (48,459 - 88,350) | (129,678 - 202,674) |
| Gout | 61,612 | 131,704 | 193,315 |  |  |  | 24,035 | 53,057 | 77,092 |
|  | (39,374 - 85,360) | (71,487 - 202,822) | (128,864 - 265,842) |  |  |  | (15,084 - 33,746) | (28,162 - 82,779) | (50,581 - 107,181) |
| **Other diseases** |  |  |  |  |  |  |  |  |  |
| Gallbladder and biliary diseases | 147,656 | 24,629 | 172,286 | 4,161 | 1,223 | 5,384 | 61,795 | 9,775 | 71,570 |
|  | (116,656 - 182,913) | (16,061 - 34,281) | (132,862 - 216,942) | (3,243 - 5,216) | (774 - 1,723) | (4,026 - 6,927) | (48,343 - 77,023) | (6,280 - 13,715) | (54,740 - 90,637) |
| Asthma | 161,827 | 78,145 | 239,971 | 3,715 | 1,480 | 5,194 | 88,042 | 41,507 | 129,548 |
|  | (112,866 - 219,081) | (55,742 - 104,245) | (168,795 - 323,133) | (2,491 - 5,142) | (1,032 - 2,006) | (3,521 - 7,133) | (60,490 - 120,290) | (29,208 - 56,092) | (89,961 - 176,217) |
| Alzheimer's disease and other dementias | 24,868 | 7,256 | 32,124 | 1 | 0 | 1 | 11,377 | 3,507 | 14,884 |
|  | (2,919 - 49,467) | (1,334 - 13,689) | (4,432 - 63,088) | (0 - 1) | (0 - 0) | (0 - 2) | (-2,197 - 26,183) | (-10 - 7,306) | (-2,131 - 33,337) |
| Cataract | 29,429 | 12,193 | 41,623 |  |  |  | 13,770 | 6,231 | 20,002 |
|  | (12,946 - 47,658) | (5,363 - 19,582) | (18,184 - 67,233) |  |  |  | (547 - 27,924) | (1,174 - 11,781) | (2,192 - 39,518) |

UI: uncertainty interval, n: mean number ^#^Musculoskeletal diseases and cataract are not linked to disease specific mortality.

# References

1. National Council for, P., et al., *Kenya Demographic and Health Survey 1993*. 1994, NCPD, CBS, and Macro International: Calverton, Maryland, USA.

2. National Council for, P., et al., *Kenya Demographic and Health Survey 1998*. 1999, NDPD, CBS, and Macro International: Calverton, Maryland, USA.

3. Central Bureau of Statistics, C.B.S.K., M.O.H.K. Ministry of Health, and O.R.C. Macro, *Kenya Demographic and Health Survey 2003*. 2004, CBS, MOH, and ORC Macro: Calverton, Maryland, USA.

4. Kenya National Bureau of Statistics, K., et al., *Kenya Demographic and Health Survey 2008-09*. 2010, KNBS and ICF Macro: Calverton, Maryland, USA.

5. Kenya National Bureau of, S., et al., *Kenya Demographic and Health Survey 2014*. 2015: Rockville, MD, USA.

6. World Health Organization. *NCD microdata repository: 2015 Kenya STEPwise approach to Surveillance of NCD risk factors (STEPS) survey*. 2018 [cited 2020 November ].

7. Ministry of Health Republic of Kenya, *Kenya STEPwise Survey for Non-Communicable Diseases Risk Factors 2015 Report*. 2015, Ministry of Health Division of Non Communicable Diseases, Kenya National Bureau of Statistics, World Health Organization.

8. Greger, M., *A Whole Food Plant-Based Diet Is Effective for Weight Loss: The Evidence.* American journal of lifestyle medicine, 2020. **14**(5): p. 500-510.

9. Papadaki, A., E. Nolen-Doerr, and C.S. Mantzoros, *The Effect of the Mediterranean Diet on Metabolic Health: A Systematic Review and Meta-Analysis of Controlled Trials in Adults.* Nutrients, 2020. **12**(11): p. 3342.

10. Motswagole, B., et al., *The Association of General and Central Obesity with Dietary Patterns and Socioeconomic Status in Adult Women in Botswana.* Journal of Obesity, 2020. **2020**.

11. Vogliano, C., et al., *Assessing diet quality of indigenous food systems in three geographically distinct solomon islands sites (Melanesia, Pacific islands).* Nutrients, 2021. **13**(1): p. 1-21.

12. Zhu, R., et al., *Adherence to a Plant-Based Diet and Consumption of Specific Plant Foods-Associations with 3-Year Weight-Loss Maintenance and Cardiometabolic Risk Factors: A Secondary Analysis of the PREVIEW Intervention Study.* Nutrients, 2021. **13**(11): p. 3916.

13. Agnoli, C., et al., *Adherence to a Mediterranean diet and long-term changes in weight and waist circumference in the EPIC-Italy cohort.* Nutrition & diabetes, 2018. **8**(1): p. 22-22.

14. Leonetti, F., et al., *Effects of basic traditional Chinese diet on body mass index, lean body mass, and eating and hunger behaviours in overweight or obese individuals.* J Tradit Chin Med, 2016. **36**(4): p. 456-63.

15. Liguori, A., et al., *Effect of a basic Chinese traditional diet in overweight patients.* J Tradit Chin Med, 2013. **33**(3): p. 322-4.

16. Swinburn, B., et al., *The global obesity pandemic: shaped by global drivers and local environments.* Lancet, 2011. **378**(9793): p. 804-14.

17. Vorster, H.H., A. Kruger, and B.M. Margetts, *The nutrition transition in Africa: can it be steered into a more positive direction?* Nutrients, 2011. **3**(4): p. 429-441.

18. Popkin, B.M., *The nutrition transition in low-income countries: an emerging crisis.* Nutr Rev, 1994. **52**(9): p. 285-98.

19. Popkin, B.M. and S.W. Ng, *The nutrition transition to a stage of high obesity and noncommunicable disease prevalence dominated by ultra-processed foods is not inevitable.* Obesity Reviews, 2022. **23**(1): p. e13366.

20. Raschke, V., et al., *Content of a novel online collection of traditional east African food habits (1930s-1960s): data collected by the Max-Planck-Nutrition Research Unit, Bumbuli, Tanzania.* 2007. **16 1**: p. 140-51.

21. Kimenju, S.C., et al., *Do supermarkets contribute to the obesity pandemic in developing countries?* Public Health Nutrition, 2015. **18**(17): p. 3224-3233.

22. Demmler, K.M., et al., *Supermarket purchase contributes to nutrition-related non-communicable diseases in urban Kenya.* PLOS ONE, 2017. **12**(9): p. e0185148.

23. Food and Agriculture Organization (FAO), *World agriculture: towards 2015/2030. An FAO perspective*, Jelle Bruinsma, Editor. 2003, Food and Agriculture Organization of the United Nations, Rome: Earthscan Publications Ltd London.

24. NCD Risk Factor Collaboration (NCD-RisC), *Trends in adult body-mass index in 200 countries from 1975 to 2014: a pooled analysis of 1698 population-based measurement studies with 19.2 million participants.* The Lancet, 2016. **387**(10026): p. 1377-1396.

25. National Research Council (US) Subcommittee, *Recommended Dietary Allowances*, in *Recommended Dietary Allowances*. 1989, National Academies Press (US), Washington (DC): <https://www.ncbi.nlm.nih.gov/books/NBK234938/>.

26. Euromonitor International, *Euromonitor from trade sources/national statistics*. 2022: <https://www.euromonitor.com/>.

27. Cawley, J. and D. Frisvold, *The Incidence of Taxes on Sugar-Sweetened Beverages: The Case of Berkeley, California (August 2015): National Bureau of Economic Research Working Paper No. w21465*. 2015: SSRN: <https://ssrn.com/abstract=2645561>.

28. Andreyeva, T., et al., *Outcomes Following Taxation of Sugar-Sweetened Beverages: A Systematic Review and Meta-analysis.* JAMA Network Open, 2022. **5**(6).

29. Cabrera Escobar, M.A., et al., *Evidence that a tax on sugar sweetened beverages reduces the obesity rate: a meta-analysis.* BMC Public Health, 2013. **13**(1): p. 1072.

30. Global Dietary Database, *2018 Global Dietary Intake Estimates*. 2022, Global Dietary Database and Gerald J. and Dorothy R. Friedman School of Nutrition Science and Policy at Tufts University: <https://www.globaldietarydatabase.org/>.

31. Department of Health and Human Services Food and Drug Administration, *Food Labeling: Nutrition Labeling of Standard Menu Items in Restaurants and Similar Retail Food Establishments Final Regulatory Impact Analysis FDA–2011–F–0172*, Office of Regulations Policy and Social Sciences Center for Food Safety and Applied Nutrition, Editor. 2014.

32. Queensland Government, *Fast Choices: kilojoule menu labelling scheme - Food Act 2006* Food Safety Standards and Regulation Department of Health, Editor. 2017, State of Queensland (Queensland Health): [www.health.qld.gov.au](file:///C:\Users\s5097420\Downloads\www.health.qld.gov.au).

33. Zlatevska, N., N. Neumann, and C. Dubelaar, *Mandatory Calorie Disclosure: A Comprehensive Analysis of Its Effect on Consumers and Retailers.* Journal of Retailing, 2017. **94**: p. 89-101.

34. World Health Organization, *Scaling up action against noncommunicable diseases: how much will it cost?* 2011: Geneva: World Health Organization.

35. Republic of Kenya, *Kenya National food and nutrition security policy implementation framework 2017-2022*, Ministry of Agriculture Livestock and Fisheries, Editor. 2017: Kilimo House, Nairobi, Kenya.

36. The World Bank Group. *Official exchange rate (Local Currency Unit per US Dollar, period average)*. 2022; Available from: <https://data.worldbank.org/indicator/PA.NUS.FCRF?locations=KE>.

37. Subramanian, S., et al., *Cost and affordability of non-communicable disease screening, diagnosis and treatment in Kenya: Patient payments in the private and public sectors.* PLOS ONE, 2018. **13**(1): p. e0190113.

38. Atieno, O.M., et al., *Pilot study assessing the direct medical cost of treating patients with cancer in Kenya; findings and implications for the future.* Journal of Medical Economics, 2018. **21**(9): p. 878-887.

39. Aminde, L.N., et al., *Estimation and determinants of direct medical costs of ischaemic heart disease, stroke and hypertensive heart disease: evidence from two major hospitals in Cameroon.* BMC Health Services Research, 2021. **21**(1): p. 140.

40. Gaziano, T.A., et al., *Hypertension education and adherence in South Africa: a cost-effectiveness analysis of community health workers.* BMC Public Health, 2014. **14**(1): p. 240.

41. GBD Chronic Kidney Disease Collaboration, *Global, regional, and national burden of chronic kidney disease, 1990–2017: a systematic analysis for the Global Burden of Disease Study 2017.* The Lancet, 2020. **395**(10225): p. 709-733.

42. Subramanian, S., et al., *Cost-effectiveness of risk stratified medication management for reducing premature cardiovascular mortality in Kenya.* PLOS ONE, 2019. **14**(6): p. e0218256.

43. White, A.D., et al., *Community surveillance of coronary heart disease in the Atherosclerosis Risk in Communities (ARIC) Study: Methods and initial two years' experience.* Journal of Clinical Epidemiology, 1996. **49**(2): p. 223-233.

44. Perman, G., et al., *Cost-effectiveness of a hypertension management programme in an elderly population: a Markov model.* Cost Effectiveness and Resource Allocation, 2011. **9**(1): p. 4.

45. Global Burden of Disease Collaborative Network, *Global Burden of Disease Study 2019 (GBD 2019) Population Estimates 1950-2019*. 2020, Seattle, United States of America: Institute for Health Metrics and Evaluation (IHME).

46. Drummond, M.F., et al., *Methods for the economic evaluation of health care programmes*. Fourth ed. 2015, New York, United States of America: Oxford University Press. 43-43.

47. Nomaguchi, T., et al., *The impact on productivity of a hypothetical tax on sugar-sweetened beverages.* Health Policy, 2017. **121**(6): p. 715-725.

48. Kenya National Bureau of Statistics, *Economic Survey 2021*. 2021: Nairobi, Kenya. <https://www.knbs.or.ke/>.

49. Salomon, J.A., et al., *Disability weights for the Global Burden of Disease 2013 study.* The Lancet Global Health, 2015. **3**(11): p. e712-e723.
